# Microfluidic-based production of [^68^Ga]Ga-FAPI-46 and [^68^Ga]Ga-DOTA-TOC using the cassette-based iMiDEV™ microfluidic radiosynthesizer

**DOI:** 10.1186/s41181-023-00229-9

**Published:** 2023-12-13

**Authors:** Hemantha Mallapura, Olga Ovdiichuk, Emma Jussing, Tran A. Thuy, Camille Piatkowski, Laurent Tanguy, Charlotte Collet-Defossez, Bengt Långström, Christer Halldin, Sangram Nag

**Affiliations:** 1 Department of Clinical Neuroscience, Center for Psychiatry Research, Karolinska Institutet and Stockholm County Council, SE-17176 Stockholm, Sweden; 2 Nancyclotep, Molecular Imaging Platform, 5 Rue du Morvan, 54500 Vandoeuvre Les Nancy, France; 3 Department of Oncology and Pathology, Karolinska Institutet, 17177 Stockholm, Sweden; 4 Department of Radiopharmacy, Karolinska University Hospital, 17176 Stockholm, Sweden; 5 PMB-Alcen, Route des Michels CD56, 13790 Peynier, France; 6 Inserm, IADI, Université de Lorraine, 54000 Nancy, France; 7 Department of Chemistry, Uppsala University, 75123 Uppsala, Sweden

**Keywords:** Positron emission tomography (PET), Radiopharmaceuticals, Microfluidics, iMiDEV, [^68^Ga]Ga-FAPI-46, [^68^Ga]Ga-DOTA-TOC, Radiolabeling, Dose-on-demand (DOD), Radiotracers

## Abstract

**Background:**

The demand for ^68^Ga-labeled radiotracers has significantly increased in the past decade, driven by the development of diversified imaging tracers, such as FAPI derivatives, PSMA-11, DOTA-TOC, and DOTA-TATE. These tracers have exhibited promising results in theranostic applications, fueling interest in exploring them for clinical use. Among these probes, ^68^Ga-labeled FAPI-46 and DOTA-TOC have emerged as key players due to their ability to diagnose a broad spectrum of cancers ([^68^Ga]Ga-FAPI-46) in late-phase studies, whereas [^68^Ga]Ga-DOTA-TOC is clinically approved for neuroendocrine tumors. To facilitate their production, we leveraged a microfluidic cassette-based iMiDEV radiosynthesizer, enabling the synthesis of [^68^Ga]Ga-FAPI-46 and [^68^Ga]Ga-DOTA-TOC based on a dose-on-demand (DOD) approach.

**Results:**

Different mixing techniques were explored to influence radiochemical yield. We achieved decay-corrected yield of 44 ± 5% for [^68^Ga]Ga-FAPI-46 and 46 ± 7% for [^68^Ga]Ga-DOTA-TOC in approximately 30 min. The radiochemical purities (HPLC) of [^68^Ga]Ga-FAPI-46 and [^68^Ga]Ga-DOTA-TOC were 98.2 ± 0.2% and 98.4 ± 0.9%, respectively. All the quality control results complied with European Pharmacopoeia quality standards. We optimized various parameters, including ^68^Ga trapping and elution, cassette batches, passive mixing in the reactor, and solid-phase extraction (SPE) purification and formulation. The developed synthesis method reduced the amount of precursor and other chemicals required for synthesis compared to conventional radiosynthesizers.

**Conclusions:**

The microfluidic-based approach enabled the implementation of radiosynthesis of [^68^Ga]Ga-FAPI-46 and [^68^Ga]Ga-DOTA-TOC on the iMiDEV™ microfluidic module, paving the way for their use in preclinical and clinical applications. The microfluidic synthesis approach utilized 2–3 times less precursor than cassette-based conventional synthesis. The synthesis method was also successfully validated in a similar microfluidic iMiDEV module at a different research center for the synthesis of [^68^Ga]Ga-FAPI-46 with limited runs. Our study demonstrated the potential of microfluidic methods for efficient and reliable radiometal-based radiopharmaceutical synthesis, contributing valuable insights for future advancements in this field and paving the way for routine clinical applications in the near future.

**Supplementary Information:**

The online version contains supplementary material available at 10.1186/s41181-023-00229-9.

## Introduction

The gallium-68 (^68^Ga) radionuclide has received considerable attention due to its availability via a ^68^Ge/^68^Ga generator and fast and efficient ^68^Ga-complexation chemistry (Breeman et al. 2011). As a result, various ^68^Ga-radiotracers have been introduced in the field of PET imaging for the diagnosis of oncological diseases (Satpati 2021). The development of new radiotracers labeled with ^68^Ga-radionuclide has increased the demand for diagnostic applications (Kratochwil et al. 2019). In the meantime, synthesis methods for ^68^Ga-radiotracers have also evolved from manual radiolabeling to cassette-based automated radiolabeling using radiosynthesizers (Mueller et al. 2016; Pieve et al. 2022). Numerous automated radiosynthesizers have been developed to meet the demand for radiotracer production and enable the synthesis of PET radiotracers (Bruton and Scott 2020).

Conventional radiosynthesis methods are more suitable for large-scale centralized production; however, they require a large quantity of chemicals and reagents. In contrast, microfluidic techniques have several advantages over conventional methods and are suitable for decentralized production. Due to the microscale volume, microfluidic techniques achieve a larger surface-area-to-volume ratio, leading to a faster reaction rate and shorter reaction time in radiosynthesis (Gillies et al. 2006; Lu and Pike 2007) and are viable for dose-on-demand (DOD) production (Arima et al. 2013; Uz Zaman et al. 2014). Recently, research has focused on implementing microfluidic techniques for the radiosynthesis of PET radiotracers (Pascali et al. 2013; Knapp et al. 2020).

Many prototypes of microfluidic modules have been demonstrated, and several microfluidic techniques have been applied in producing PET radiotracers (Elkawad et al. 2022). These are suitable for manufacturing DODs in preclinical and clinical settings. Nevertheless, incorporating these microfluidic methods into routine production remains challenging due to reproducibility issues, and GMP compliances mainly because most of them are custom-made for specific applications and associated engineering problems (Knapp et al. 2020). For radiotracer synthesis, continuous flow microfluidics (Wilson et al. 2000; Matesic et al. 2017), batch-type microfluidics, and digital microfluidics have been investigated (Lebedev et al. 2013; Wang et al. 2019). However, the implementation of microfluidic techniques in all kinds of radiotracer production is ongoing, and reliable microfluidic radiosynthesizers are needed to utilize their advantages in routine preclinical and clinical environments.

The iMiDEV™ batch-type microfluidic radiosynthesizer was investigated for the synthesis of fluorine-18 (^18^F), carbon-11 (^11^C), and ^68^Ga-radiotracers at room temperature (Ovdiichuk et al. 2021, 2022; Mallapura et al. 2022) as well as at elevated temperature (Ovdiichuk et al. 2023). The radiolabeling of DOTA chelators with the ^68^Ga-radionuclide requires high temperatures (León-Rodríguez and Kovacs 2008). Currently, most conventional methods use cassette-based approaches to synthesize ^68^Ga-radiotracers, which requires 40–50 µg (Nelson et al. 2022; Alfteimi et al. 2022) of precursors for radiolabeling. The amount of precursor can be reduced by applying microfluidic-based synthesis. A low peptide quantity is recommended for producing receptor-targeted radiotracers because higher peptide amounts could cause receptor saturation and reduced uptake in the targeted receptors (Breeman et al. 2001). Microfluidic techniques have demonstrated the viability of this approach for radiotracer synthesis, leading to the production of radiopharmaceuticals with high molar activity (Wang and Dam 2020).

We selected to synthesize well-known radiotracers such as ^68^Ga-labeled FAPI-46 and DOTA-TOC. [^68^Ga]Ga-FAPI-46 is utilized for the imaging of fibroblast activation protein in cancerous tissues (Kratochwil et al. 2019), while [^68^Ga]Ga-DOTA-TOC is primarily used for imaging neuroendocrine tumors (Hennrich and Benešová 2020).

In this study, we explored the production of [^68^Ga]Ga-FAPI-46 and [^68^Ga]Ga-DOTA-TOC by using a microfluidic cassette-based iMiDEV™ radiosynthesizer. We aimed to (1) optimize the elution of [^68^Ga]GaCl_3_ from reactor 1 (R1) filled with a cation exchanger to improve the recovery of Ga-68, (2) increase the radiochemical conversion by improving the mixing of reagents in reactor 2 (R2) using a passive mixing technique, and iii) transfer and validate the synthesis method using the iMiDEV™ radiosynthesizer at a different research center under identical experimental conditions.

## Results

Radionuclide extraction, radiolabeling, and purification were performed on embedded microreactors on the cassette. For initial tests, 50 to 200 MBq radioactivity was used, and after optimization of radiolabeling, 1100 MBq to 1200 MBq was used to produce a single clinical dose. Manual radiolabeling was performed to adjust the pH of the reaction mixture, and the radiochemical yield (non-isolated) of manual syntheses were 95 ± 5% (n = 4).

The detailed production of radiotracers is summarized in Table 1. The amounts of final products for [^68^Ga]Ga-FAPI-46 and [^68^Ga]Ga-DOTA-TOC were 381 ± 31 MBq and 367 ± 78 MBq, respectively. The radiochemical yield_dc_ (RCY_dc_) of [^68^Ga]Ga-FAPI-46 was 44 ± 5% (n = 3), with radiochemical purities of 97.7 ± 0.5% (HPLC) and 98.2 ± 0.15% (TLC). For [^68^Ga]Ga-DOTA-TOC, RCY_dc_ was 46 ± 7% (n = 3) with radiochemical purities of 99.6 ± 0.2% (HPLC) and 99.1 ± 0.7% (TLC). The apparent molar activity (AMA) of [^68^Ga]Ga-FAPI-46 and [^68^Ga]Ga-DOTA-TOC at the end of synthesis were 30 ± 11 GBq/µmol (n = 3) and 61 ± 10 GBq/µmol (n = 3), respectively. The details of the quality control tests are summarized in Table 2. All performed quality control tests complied with European Pharmacopoeia (Ph. Eur.) quality standards. The chromatograms of TLC and HPLC (Additional file 1: Figure S1-S4) and the reaction schematic of [^68^Ga]Ga-DOTA-TOC and [^68^Ga]Ga-FAPI-46 (Additional file 1: Figure S5-S6) are found in the Additional file 1.

**Table 1:** Production summary of [^68^Ga]Ga-FAPI-46 and [^68^Ga]Ga-DOTA-TOC

Parameters	[^68^Ga]Ga-FAPI-46	[^68^Ga]Ga-DOTA-TOC
No of synthesis (n)	3	3
Starting activity (MBq)	1219 ± 35	1110 ± 78
Precursor mass (µg)	20	20
Final product (MBq)	381 ± 31	367 ± 78
Decay corrected RCY (%)	44 ± 5	46 ± 7
Apparent molar activity (GBq/µmol)	30 ± 11	61 ± 10

**Table 2:** Summary of QC tests of [^68^Ga]Ga-FAPI-46 and [^68^Ga]Ga-DOTA-TOC

Test	Acceptance criteria	[^68^Ga]Ga-FAPI-46	[^68^Ga]Ga-DOTA-TOC
Appearance	Clear or slightly yellow and free of particles	Conform	Conform
pH	4.0–8.0	5.83 ± 0.1	5.83 ± 0.1
Total radiochemical purity^a^ (%)	≥ 91	96.0 ± 0.40	97.6 ± 1.20
Filter integrity (bar)	≥ 3.5	4.1 ± 0.1	4.2 ± 0.1
Radionuclide identity by half-life (min)	62–74	67.5 ± 0.3	68 ± 0.3
Bacterial endotoxins (EU/mL)	< 17.5	< 5.0	< 5.0
Ethanol (%) G.C	< 10	6.7 ± 0.2	6.1 ± 0.3
Radiochemical stability after 3 h (%)	≥ 91	95.8 ± 0.80	97.8 ± 0.60

^a^(100-A)*B; A = impurity on iTLC (unbound ^68^Ga) and B = RCP on HPLC/100 G.C.- Gas Chromatography

In order to verify the reproducibility of the complete automated synthesis, it was repeated at Nancyclotep (radiochemistry laboratory) using the same experimental settings as the validation runs. The pulse flow approach allowed us to obtain [^68^Ga]Ga-FAPI-46 with 42 ± 6% (n = 3) radiochemical yield and high radiochemical purity (≥ 95%). The activity losses in the cassette and module were 11 ± 2% (n = 3). All the details are summarized in Additional file 1: Table S1.

## Discussion

**Fig. 1:**
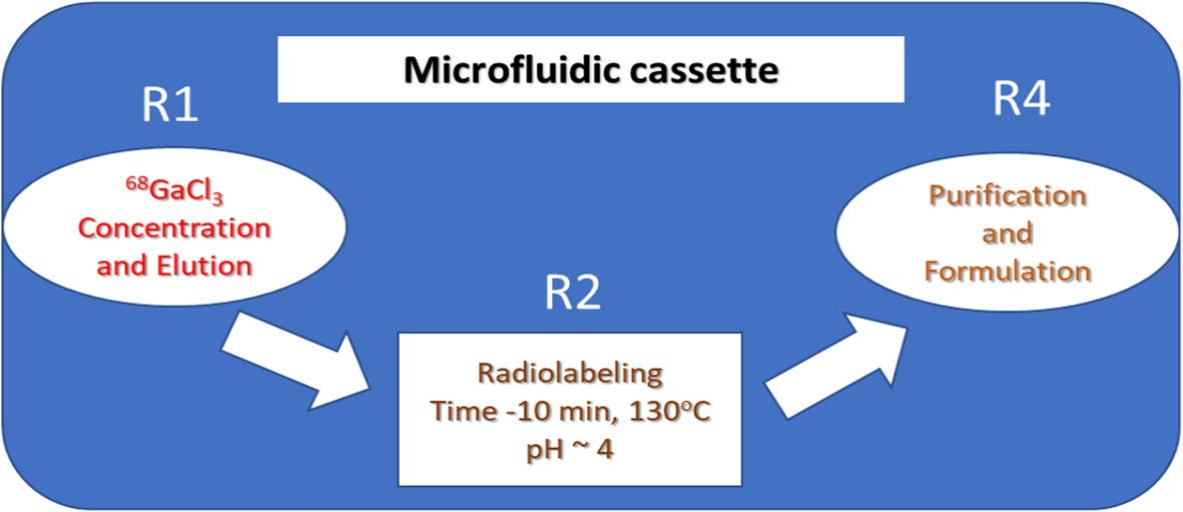
Radiosynthesis steps performed on the cassette

The radiosynthesis optimization was performed in three steps, as shown in Fig. 1. To optimize [^68^Ga]Ga^3+^ concentration, Chromafix PS-H + resin was selected, and different parameters, such as direct and reverse trapping and elution, and different batches of cassettes were evaluated. For radiolabeling, different mixing techniques were explored for good mixing of [^68^Ga]Ga^3+^ and a buffer-peptide mixture on R2 to achieve a high radiochemical yield. After optimization, HLB and C18 resins were examined for SPE purification and formulation, and product elution was assessed using different elution pressures for ethanol. Finally, the optimized experimental conditions were validated to examine the reproducibility of the results at a different research center using the same synthesis module.

### Optimization of elution of [^68^Ga]GaCl_3_

**Fig. 2:**
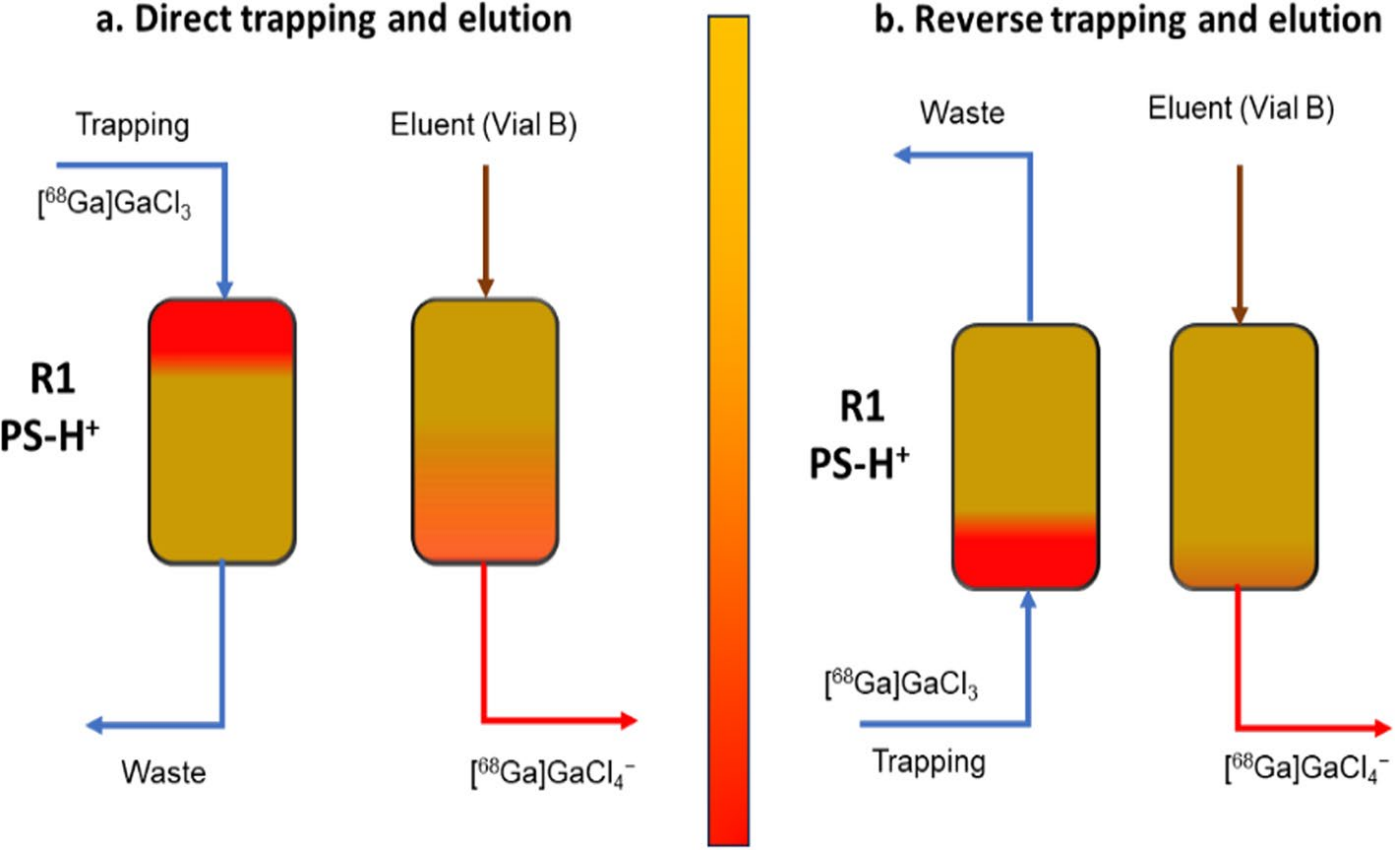
Trapping and elution of [^68^Ga]GaCl_3_; **a** Direct trapping and **b** Reverse trapping

The concentration and elution of [^68^Ga]GaCl_3_ are critical steps in achieving a high radiolabeling yield when using a generator for the ^68^Ga-eluate. However, the limited volume of reactor R2 (286 µL) poses a challenge, as it restricts the volume of eluent available for elution, along with the buffer-peptide mixture required for the radiolabeling process. Different eluent concentrations and volumes were tested for direct trapping and elution of [^68^Ga]GaCl_3_ to improve the recovery from R1. Considering the volume of R2, the initial volume of the eluent was 170 µL, and the concentration was 0.12 M HCl in 5 M NaCl (eluent 1) (Jussing et al. 2021). The elution pressure on vial B (glass vial; 0.3 mL) for all tests was 0.2 bar. The trapping efficiency for all the tests was > 98.5%. The concentration and elution results of [^68^Ga]GaCl_3_ are summarized in Table 3. The trapping and elution of [^68^Ga]GaCl_3_ in the same direction are shown in Fig. 2a. The recovery of [^68^Ga]Ga^3+^ from the direct trapping was 78 ± 4% (n = 10). Despite achieving more than 80% recovery in some cases, it lacked reproducibility, and occasionally, we observed a low recovery of [^68^Ga]GaCl_3_ (45 ± 20%; n = 6), resulting in a significant loss of radioactivity that adversely affected the final radiochemical yield. The fluctuation in the recovery of [^68^Ga]GaCl_3_ was attributed to the larger interaction surface area and variations in the density of the bead filling on R1. The trapping and elution direction on R1 was changed to address this issue, and the eluent concentration was adjusted to 0.15 M HCl in 5 M NaCl (eluent 2). In addition, different eluent volumes were explored to optimize the recovery process*.*

**Table 3:** Summary of concentration and elution of [^68^Ga]GaCl_3_

Trapping and elution	Mixing technique	No of syn (n)	Volume (µL)	Conc of eluent	Recovery (%)
Direct trapping	Normal flow	10	170	Eluent 1	78 ± 4
Reverse trapping	Normal flow	12	140	Eluent 2	84 ± 4
Reverse trapping	Continuous flow	21	140	Eluent 2	75 ± 8
Reverse trapping	Alternative flow	6	120	Eluent 2	79 ± 6
Reverse trapping	Pulse flow	17	140	Eluent 2	88 ± 4
Reverse trapping	Pulse flow	4	200	Eluent 2	91 ± 6

The reverse trapping and elution (Fig. 2b) with the normal and pulse flows yielded 84 ± 4% (n = 12) and 88 ± 4% (n = 17) of recovery with 140 µL of eluent 2. The recovery with continuous and alternative flow was 75 ± 8% (n = 21) and 79 ± 6% (n = 6), respectively. The high recovery with reverse trapping and elution was explained by the low residual activity left on R1 because of the lower surface area interaction of the resins with radioactivity when [^68^Ga]Ga^3+^ eluted from R1 (Fig. 2b). The volume of eluent 2 was increased to 200 µL for radiolabeling optimization, which slightly increased the recovery to 91 ± 6% (n = 4). With the reverse trapping implementation, the recovery of [^68^Ga]Ga^3+^ increased by almost 20%.

Applying different pressures to trap [^68^Ga]GaCl_3_ on Chromafix PS-H + resin revealed no significant difference between 0.75 and 1.0 bar using pulse flow elution. Both pressure values resulted in a recovery rate of over 85% with the pulse flow. Therefore, we used 1.0 bar for trapping [^68^Ga]GaCl_3_ and 200 mbar for elution in all subsequent tests performed during the radiolabeling optimization process. However, despite the optimization of elution conditions, lower recovery rates were observed with various batches of cassettes due to variations in the density of the beads on R1. These differences in bead density were observed among the different batches of cassettes utilized in the synthesis. The bead density was measured using pressure drop tests on R1. The highest recovery was achieved when the pressure drop on R1 fell within the range of 0.28–0.30 bar when 1 bar was applied on R1, while lower pressure drop values below 0.28 bar resulted in reduced recovery rates. Notably, the cassettes used in this study consistently exhibited pressure drops of approximately 0.28–0.30 bar.

### Optimization of radiolabeling

#### Mixing techniques

After the elution of [^68^Ga]Ga^3+^ was optimized, radiolabeling was performed for FAPI-46 using different mixing techniques to achieve a high radiolabeling yield. Radiolabeling was performed manually using the same volume of eluent, acetate buffer, and peptide to adjust the pH of the reaction mixture. The radiochemical yields (non-isolated) obtained by applying different mixing techniques and using different volumes of eluent and buffer-peptide mixtures are summarized in Table 4.

**Table 4:** Summary of radiochemical yield (non-isolated) with different mixing techniques using iMiDEV™ module

Trapping and elution	Mixing technique	No of synthesis (n)	Eluent (µL)	Buffer volume (µL)	Peptide (µg)	Radiochemical yield_dc_ (%)**
Direct trapping	Normal flow	8	170*	110	20	64 ± 16
Reverse trapping	Normal flow	12	170	110	20	27 ± 28
Reverse trapping	Continuous flow	16	140	120	20	67 ± 16
Reverse trapping	Alternative flow	6	120	160	20	68 ± 18
Reverse trapping	Pulse flow	4	200	150	20	79 ± 8

*Eluent 1

**Non-isolated yield

First, radiolabeling with direct trapping and normal flow was investigated. Radiolabeling was challenging because of the inadequate mixing of [^68^Ga]Ga^3+^ and the buffer-peptide mixture; despite this, the achieved radiochemical yield (non-isolated) was 64 ± 16% (n = 8). The inadequate mixing of the buffer-peptide mixture with radioactivity in R2 was primarily attributed to slow diffusion rates, which resulted from the viscosity of the solvent, bubbles formation, and laminar behavior of fluids in the microfluidic cassette.

The trapping direction (reverse trapping) of [^68^Ga]GaCl_3_ and eluent concentration (eluent 2) were changed to improve the recovery and radiochemical yield. Although a good recovery (Table 3) was obtained, this combination resulted in a poor radiochemical yield (non-isolated) of 27 ± 28% (n = 12) for normal flow mixing. This can be explained by the quick elution of concentrated [^68^Ga]Ga^3+^ into R2, which did not mix with the buffer-peptide mixture, resulting in poor radiolabeling yield.

The continuous flow with reverse trapping and elution of the [^68^Ga]Ga^3+^ and buffer-peptide mixture was examined to improve radiochemical yield. This approach worked better with reverse trapping, giving a radiochemical yield (non-isolated) of 67 ± 16% (n = 16). We encountered difficulties in ensuring reproducibility, primarily arising from the mixing issue during the elution of [^68^Ga]Ga^3+^. The elution process relied on a pressure-driven mechanism, which became challenging to maintain consistently as different cassette batches were used. The pressure drop variations within each batch necessitated careful management of the pressure release and drop in R1, which proved impractical for every test. Even minor adjustments in flow restrictions directly impacted mixing, causing the buffer to be pushed ahead of [^68^Ga]Ga^3+^ and subsequently leading to poor radiolabeling yields.

The alternative flow with reverse trapping and elution was evaluated to address the mixing problem in R2. This method did not substantially enhance the radiochemical yield (non-isolated; 68 ± 18%; n = 6) owing to bubble formation impacting the mixing. During most of the syntheses, the first portion of the buffer was transferred to the end of R2 while [^68^Ga]Ga^3+^ was eluted from R1, which caused the blockage of a vent between V13 and V14 and prevented the flow of reagents from vials B and C.

**Fig. 3:**
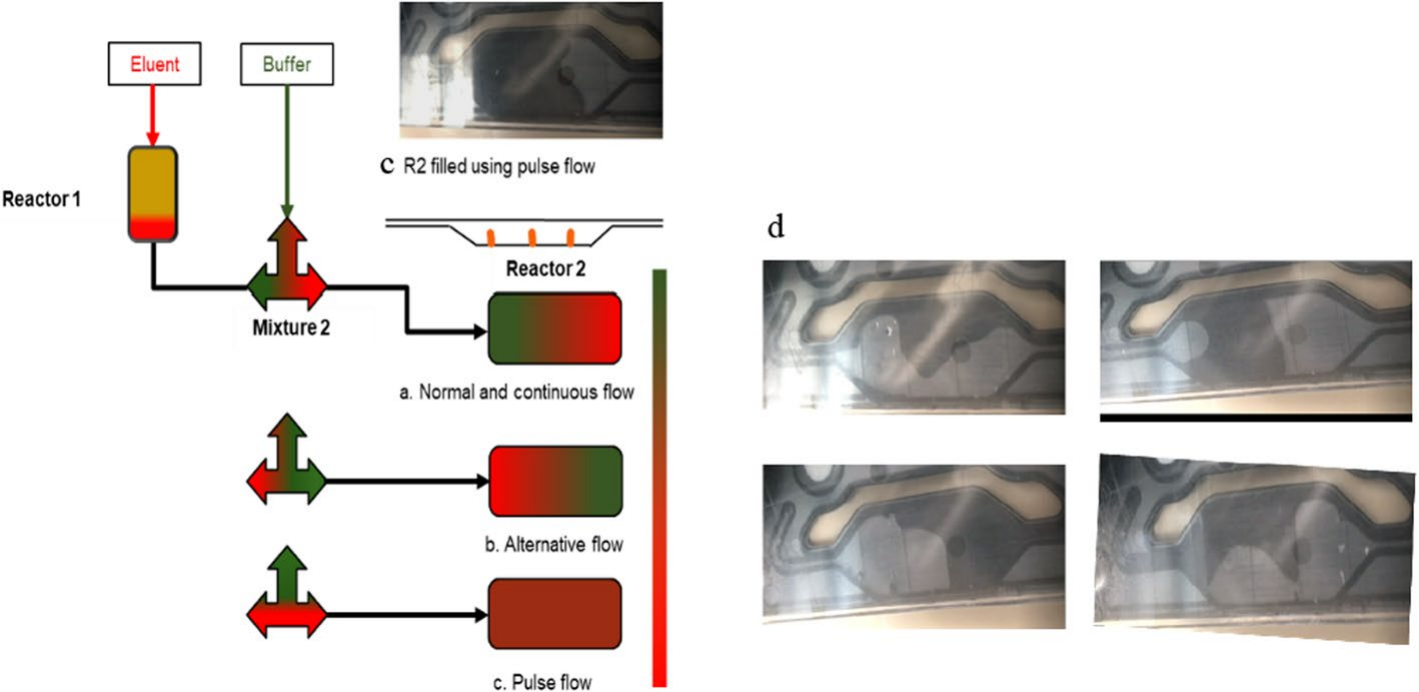
Graphical representation of elution of [^68^Ga]GaCl_3_ and mixing with buffer-peptide mixture on R2; **a** [^68^Ga]Ga ^3+^ eluted quickly before buffer (Normal and continuous flow); **b** Buffer eluted quickly before [^68^Ga]Ga ^3+^ (Alternative flow); **c** [^68^Ga]Ga^3+^ and buffe mixing (Pulse flow); **d** Insufficient filling of R2 with various mixing techniques and formation of bubbles

Partial elution of [^68^Ga]Ga^3+^ on R1 was performed to release 30–40% of the trapped [^68^Ga]GaCl_3_ on R1 prior to mixing with the buffer-peptide mixture in M2. A graphical representation of the filling of reagents on R2 using different flow/mixing techniques and pictures of insufficient filling of R2 are shown in Fig. 3. The impact of the rapid movement of buffer and eluent on the mixing is depicted in Fig. 3a, b (left side). Either the buffer or radioactivity went to the other end of R2 due to slow/quick elution. Normal, continuous, and alternative flows for the mixing technique are more prone to form bubble/s on R2 (Fig. 3d; right side), thereby lowering the radiolabeling yield. In contrast, the pulse flow technique demonstrated excellent mixing of reagents, as is evident from the filled R2 in the microfluidic cassette, as shown in Fig. 3c (top left).

When the bubble formed in R2, there was less surface area for the interaction between [^68^Ga]Ga^3+^ and the buffer-peptide mixture; thus, a suitable pH of the reaction mixture was not achieved. More residue of radioactivity was left on R4 when inadequate mixing was observed in R2, implicating the formation of ^68^Ga-colloid due to the variation in pH (Brom et al. 2016; McInnes et al. 2017) in R2.

Overall, while the continuous and alternative flow methods yielded comparable and satisfactory radiochemical yields, they showed limitations in terms of reproducibility. However, the alternative flow technique showed promising results despite some fluctuations in the radiolabeling yield and increased recovery compared with continuous flow. We developed and adopted a pulse flow method to further enhance this approach.

The pulse flow (continuous-alternative flow) mixing technique was adapted by optimizing the alternative flow to address mixing issues and bubble formation in R2. A suitable pH for radiolabeling was achieved because of slow mixing. Therefore, the radiochemical yield (non-isolated product) was 79 ± 8% (n = 4). Preheating of R2 was performed to increase the diffusion rate of the reagents on R2. Preheating temperatures of 50 and 70 °C (set point) were evaluated with pulse flow, and no considerable influence was observed on the final radiochemical yield. The pulse flow technique allowed high radiochemical yields and reproducibility. It also ensures consistent and dependable radiochemical yield by addressing mixing and bubble formation problems from the slow elution of [^68^Ga]Ga^3+^ and the buffer-peptide mixture, as well as with different cassette batches.

The reagents mixing issue arose mainly from the insufficient filling of R2 due to random bubble formation during the reagent mixing using different flows. We owe this to the quick/slow elution of the reagent in vial B due to variations in the pressure drop on R1 and occasionally flawed or compromised vents. The advantages and disadvantages of different mixing approaches are summarized in Table 5.

**Table 5:** Summary of pros and cons of mixing techniques

Mixing technique	Pros	Cons
Normal flow	Simple setup and procedure	Poor mixing due to bubble formation
	Easy to implement	Poor or no radiolabeling achieved
Continuous flow	Good radiolabeling in some cases	High back pressure on R1 affects mixing
	Continuous mixing of reagents in R2	Reproducibility issue with various batches of cassette
Alternative flow	Average to good radiolabeling yield	Poor mixing and bubble formation in R2
	Partial elution and addition of buffer mixture	Issues with filling R2
Pulse flow	Achieved reproducibility and high radiolabeling yield	Requires precise timing for opening MFVs
	Increased surface area for reagent interaction	Careful control needed to avoid bubble formation

#### Reaction temperature, time, and precursor amount

Initially, the reaction temperature for radiolabeling was set to 100 °C, and numerous syntheses were conducted under this condition. However, after conducting heating element studies to compare the set value with the actual temperature value inside R2, it became evident that there was a significant variation between the two values. This discrepancy was attributed to the heat transfer between the heating component and the R2 chamber, which was further confirmed through a simulation study (Ovdiichuk et al. 2023). The actual temperature inside the R2 chamber was approximately 70% of the set value. Based on these findings, the reaction temperature was increased to 130 °C to ensure a suitable temperature for effective radiolabeling.

The radiochemical yield was evaluated by assessing the impact of different reaction times. A reaction time of 5 min was chosen considering the reaction volume. However, this resulted in a low radiochemical yield (non-isolated) (50%; n = 1). Subsequently, the reaction time was extended to 10 min, resulting in a significant improvement in the radiolabeling yield of up to 86%. Consequently, the 10-min reaction time was consistently maintained for all subsequent syntheses.

Various amounts of the precursor, ranging from 10 to 40 µg, were utilized in the reaction to evaluate its effect on the radiochemical yield using the cassette. The radiochemical yields of [^68^Ga]Ga-FAPI-46 and [^68^Ga]Ga-DOTA-TOC obtained using different amounts of precursor are summarized in Table 6. Initially, a test with 10 µg of precursor yielded quantitative results in manual synthesis (n = 1). However, when the same amount was applied to the microfluidic cassettes, the radiochemical yields of [^68^Ga]Ga-FAPI-46 (31 ± 2%; decay corrected; n = 2) and [^68^Ga]Ga-DOTA-TOC (26 ± 4%; decay corrected; n = 2) was not satisfactory because of the underutilization of the precursor caused by the residual dead volume in the vial. The precursor amount was then increased from 10 µg to 40 µg to enhance the radiochemical conversion and reproducibility. When the amount of precursor increased, the radiochemical yield increased for both FAPI-46 and DOTA-TOC. Nevertheless, poor mixing also contributed to the suboptimal radiochemical yield of [^68^Ga]Ga-FAPI-46 (53 ± 1%; decay corrected; n = 2) and [^68^Ga]Ga-DOTA-TOC (41 ± 5%; decay corrected; n = 2) even though 40 µg of the precursor was used for the synthesis. After optimization of the mixing techniques, the pulse flow method was selected, and the final precursor concentration was fixed at 20 µg (2 mg/mL) for both [^68^Ga]Ga-FAPI-46 (62 ± 2%; n = 2) and [^68^Ga]Ga-DOTA-TOC (51 ± 4%; n = 2) to ensure optimal and consistent radiochemical yields. However, a total of 170 µL of buffer-precursor mixture was not used for the synthesis because of the dead volume in the precursor vial (30–50 µL), and three-fourths of the precursor amount (**≈** 15 µg) was utilized for synthesis.

**Table 6:** Summary of radiochemical yield of [^68^Ga]Ga-FAPI-46 and [^68^Ga]Ga-DOTA-TOC using different amount of precursor from the iMiDEV module

Radiotracers	Precursor (µg)	Yield_dc_ (%)
[^68^Ga]Ga-FAPI-46 (n = 2)	10	31 ± 2
	15	37 ± 1
	20	62 ± 2
	40	53 ± 1
[^68^Ga]Ga-DOTA-TOC (n = 2)	10	26 ± 4
	15	28 ± 1
	20	51 ± 4
	40	41 ± 5

#### SPE purification using HLB and C18 resins on R4

Following successful radiolabeling optimization, comprehensive radiosynthesis was conducted, incorporating solid-phase extraction (SPE) purification and formulation into the microfluidic cassette. The HLB resin was selected for the initial tests. When the product elution on R4 was performed at a pressure of 1 bar, a substantial amount of residual activity remained on the resin (as detected by the radioactivity sensor) due to rapid elution. Consequently, the elution pressure was optimized from 1 bar to 0.65 bar, resulting in improved product elution from R4 with minimal loss of product activity on R4. Subsequently, C18 resin was evaluated for SPE purification in R4.

Interestingly, no difference was observed between the two resins with respect to the product elution using 56% ethanol at the optimized elution pressure. However, when insufficient mixing occurred during the radiolabeling process, more radioactivity was retained on both resins, which was attributed to the formation of ^68^Ga-colloid due to the pH variability in the reaction mixture. HLB resin was chosen for subsequent tests in the SPE purification step, and the product was effectively eluted using a 0.65 bar elution pressure.

#### Distribution of radioactivity in the complete radiosynthesis (product, filter, and microfluidic cassette)

The radiochemical yield of [^68^Ga]Ga-FAPI-46 achieved using the pulse flow approach was 51 ± 5% (decay corrected; n = 13) (Additional file 1: Table S2). Comparing the RCY_dc_ of [^68^Ga]Ga-FAPI-46 with conventional methods 69 ± 4% (Modular-Lab Pharmtracer from Eckert and Ziegler; n = 4) (Jussing et al. 2021) and 66 ± 8% (Trasis AiO Platform; n = 10) (Pieve et al. 2022), our yields were comparable and sufficient for clinical and preclinical applications. Notably, these conventional methods also performed pre-purification of [^68^Ga]GaCl_3_. The radioactivity distribution of the complete synthesis was analyzed by measuring the radiochemical yield, synthesis waste, and filter.

**Fig. 4:**
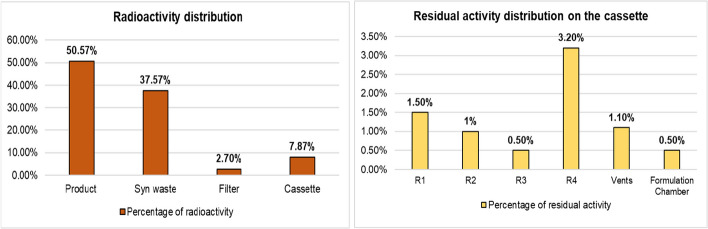
**a** Radioactivity distribution of the radiosynthesis comparing with the product, synthesis waste (syn waste), and sterile filter and the cassette. **b** Residual activity distribution on the cassettes (n = 3)

We also evaluated residual activity of the microfluidic cassette after the complete radiosynthesis. The radioactivity distribution during the complete radiosynthesis is shown in Fig. 4a. The RCY_dc_ of the successful batches of [^68^Ga]Ga-FAPI-46 and [^68^Ga]Ga-DOTA-TOC was 51 ± 4% (n = 3), synthesis waste was 38 ± 4% (n = 3), and residual activity in the sterile filter and the cassette was 3 ± 1% and 8 ± 0.1% (n = 3), respectively (Additional file 1: Table S3). More radioactivity was observed in the synthesis waste, which can be explained by the sub-optimal mixing of reagents. The cassettes were also manually sectioned to measure the individual parts to assess the residual activity distribution. A summary of the residual activity of the cassette is presented in Fig. 4b. The retained activity on R1, R2, and R4 were 1.5 ± 0.3%, 1 ± 0.6%, and 3.2 ± 1.2% (n = 3), respectively. The highest residual activity on R4 was higher due to the interaction between the large surface area of the beads and the product. Additionally, a loss of 1.1 ± 0.3% (n = 3) was observed in the vents. The activity on R3 and the formulation chamber was 0.5 ± 0.3% (n = 3) and 0.5 ± 0.1% (n = 3), respectively (Additional file 1: Table S4).

#### Apparent molar activity (AMA)

The apparent molar activities of [^68^Ga]Ga-FAPI-46 and [^68^Ga]Ga-DOTA-TOC were measured after the validation runs. The measured apparent molar activity at the end of the synthesis was 30 ± 11 GBq/µmol (n = 3) for [^68^Ga]Ga-FAPI-46 and 61 ± 10 GBq/µmol (n = 3) for [^68^Ga]Ga-DOTA-TOC. The AMA of microfluidic-based production of [^68^Ga]Ga-FAPI-46 was higher compared to kit-based production of [^68^Ga]Ga-FAPI-46 (10 ± 1.7 GBq/µmol; n = 4) (Jussing et al. 2021), and 13–30 GBq/µmol; n = 3 (Spreckelmeyer et al. 2020) and also for [^68^Ga]Ga-DOTA-TOC (21.7 ± 5.6 GBq/µmol; n = 86) (Jussing et al. 2021). This is because almost 3 times less precursor amount is utilized in the microfluidic-based synthesis. Additionally, we are working on improving radiochemical yield further to increase the AMA of ^68^Ga-labeled compounds, which are essentially applied in theranostic applications.

#### Comparison of radiochemical yield with conventional modules

The experimental condition of [^68^Ga]Ga-FAPI-46 using the iMiDEV™ microfluidic module was compared with two other conventional modules. All the details were summarized in Additional file 1: Table S5. All the quality tests requirements are comparable with conventional modules quality standard, but radiochemical yield, iMiDEV™ module (44 ± 5%; n = 3) yielded low compared to Modular-Lab Pharmtracer (MLPT) from Eckert and Ziegler (69 ± 4% n = 4) (Jussing et al. 2021) and 92 ± 8% (Trasis EasyOne; n = 3) (Alfteimi et al. 2022). However, RCYs are satisfactory for a single dose or DOD requirement. The lower yield from the iMiDEV™ could be explained by the increased synthesis time and inadequate mixing of reagents because of the bead filling of the different cassette batches. We are striving to enhance the condition of the microfluidic cassette in terms of the back pressure on R1, which is crucial for achieving reproducibility. As the transfer of all the reagents is pressure-driven, any variation in the beads density during filling attributed to their size (40–85 µm) could significantly impact small-volume transfer and mixing. Currently, we are leveraging our experience to manufacturing microfluidic cassettes with beads of uniform size. This adaptation aims to ensure consistent back pressure across the entire cassette batch, thereby eliminating variability in production from one batch to another. This principle also applies to R4, which is employed for solid phase extraction (SPE) purification, where an elevated back pressure can extend the purification duration. Eventually, this optimization could resolve the inconsistency in synthesis conditions and make the module condition robust. This indicates the potential for the implementation of the iMiDEV™ module in clinical production.

The choice of cassette material, known for its chemical insensitivity, not only upholds the structural robustness of the system but also plays a pivotal role in ensuring the integrity of the synthesis process (Ovdiichuk et al. 2021). Furthermore, there was no cross-contamination issue due to the single-use cassette, which aligns with the cassette-based production. Employing the microfluidic cassette for production carries no risk of introducing impurities during the synthesis process, ensuring product quality.

#### Validation of experimental conditions in a different research center

The same experimental conditions and synthesis recipes for the iMiDEV™ module were evaluated for the reproducibility of results in a different research center. All the reagents used in the synthesis and cassette requirements were maintained to reproduce the results. Syntheses were performed only for [^68^Ga]Ga-FAPI-46, and a radiochemical yield of 42 ± 6% (n = 3) was achieved.

The observed decrease in radiochemical yield when duplicating the setup in a different research center could stem from several factors. One critical aspect is the sensitivity of the microfluidic system to variations in environmental conditions, personal training, and equipment calibration between different laboratories. Small differences in temperature, pressure (flow dynamics), or other experimental parameters might impact the reproducibility of the synthesis. Additionally, variations in the quality and characteristics of reagents, even if seemingly identical, could contribute to differences in yields. Even minor deviations in the density of beads in reactor chambers could result in changes in flow resistance and, consequently, impact yield. Collaborative efforts between research centers to standardize protocols, calibrate equipment, and validate reagents could be crucial for enhancing the reproducibility of the microfluidic-based synthesis method across different locations. Addressing these factors systematically will contribute to the successful implementation of this technology in diverse research environments and also contribute valuable knowledge to the broader field of microfluidic-based radiotracer synthesis.

In order to bolster the resilience of the methodology and minimize the chance of errors, it is advisable to implement comprehensive training programs for staff involved in the synthesis process. Additionally, considering the potential fragility of certain components, reinforcing equipment durability, and providing clear guidelines on handling sensitive parts could contribute to minimizing the risk of failures. Robustness studies that assess the system's performance under different operator skills and potential error scenarios would be valuable in establishing the reliability of the microfluidic-based synthesis method in diverse settings. We comprehensively assess all variations from a broader perspective to address potential challenges in the validation of different synthesis methods in different research centers in the near future.

#### Limitation of the study and future perspectives

Despite the promising strides in microfluidic-based radiotracer synthesis, certain limitations merit consideration. The current focus on established tracers, such as [^68^Ga]Ga-FAPI-46 and [^68^Ga]Ga-DOTA-TOC may restrict the generalizability of findings to a broader spectrum of radiopharmaceuticals. Further exploration of the system's adaptability to diverse tracers is warranted for a comprehensive assessment. The study predominantly addresses technical aspects, necessitating additional research to evaluate economic feasibility and scalability for routine preclinical and clinical use. Fluctuations in yields underscore the need for optimization to enhance reproducibility. While efforts were made to replicate synthesis in different labs, a more extensive multi-center validation would bolster system robustness. The study does not extensively cover the challenges of regulatory approval, which is a crucial aspect of clinical implementation. Recognizing and addressing these limitations will guide future research towards a more impactful translation of microfluidic synthesis into routine clinical practice.

## Conclusions

Our study successfully optimized and established the microfluidic production of [^68^Ga]Ga-FAPI-46 and [^68^Ga]Ga-DOTA-TOC using the microfluidic cassette-based iMiDEV™ module, and achieved a sufficient radiochemical yield and high apparent molar activity for preclinical and clinical use. This method can be easily adapted to a single dose or DOD in routine preclinical and clinical applications. The pulse-flow approach for mixing effectively provided well-mixed reagents and consistent yields, leading to a fully automated process. We fine-tuned various parameters, including reaction temperature, precursor amount, and purification method. The precursor amount was optimized to 2–3 times less than that used in conventional methods. Challenges related to bead-filling variability have been addressed to enhance the yield and reproducibility. Our study demonstrated the potential of microfluidic methods for efficient and reliable radiometal-based radiopharmaceutical synthesis, contributing valuable insights for future advancements in this field, and paving the way for routine pre-clinical and also in clinical applications in the near future.

## Materials and methods

The ^68^Ge/^68^Ga generator (1.85 GBq) was purchased from Eckert and Ziegler, Berlin, Germany. Microfluidic cassettes (single-use) and automated microfluidic iMiDEV™ radiosynthesizer used in this study were supplied by PMB-Alcen, Peynier, France. Acetate buffer pH 4.6 (31048–500 mL) and water (TraceSELECT™, for trace analysis, 2.5 L) were purchased from Honeywell (Fluka), Seelze, Germany. Hydrochloric acid (HCl), ammonium acetate, methanol, trifluoracetic acid, sodium ascorbate, and 5 M NaCl were procured from Sigma Aldrich, Germany. Acetonitrile (HPLC grade) was ordered from Fisher Scientific, Sweden. DOTA-TOC (Cat. No. HY-106033) and FAPI-46 (Cat. No. HY-137331) were purchased from MedChemTronica, Sollentuna, Sweden. Sterile water (for injection) and 0.9% sodium chloride were supplied by B Braun, Melsungen, Germany. Ethanol (99.5%) was purchased from KiiltoClean, Malmö, Sweden. Ethanol absolute anhydrous was purchased from Carlo Erba Reagents SAS, Val de Reuil, France. Sterile vent filters (Millex FG. 0.2 µm, 25 mm) and sterile filters (Millex GV, 0.22 µm, 33 mm) were obtained from Merck Millipore, Carrigtwohill, Ireland. Sterile 15 mL vials were procured from Huayi Isotopes Co. (HIC), China. iTLC-SG-strips (SGI0001) were ordered from Agilent, California, USA. Milli-Q water (18 MΩ) was obtained from an in-house Millipore water purification system. Glass vials were used for reagents (4 mL and 15 mL) procured from Nordic Pack, Nykvarn, Sweden. Inserter vials (300 µL) and aluminium seals with septa (11 mm) were ordered from Thermo Scientific, Langerwehe, Germany. Wheaton® W986212NG NextGen™ V Vial® 0.3 mL Clear Glass High Recovery Vials used in syntheses were supplied by Wheaton, USA**.**

### Microfluidic cassette-based iMiDEV™ radiosynthesizer

**Fig. 5:**
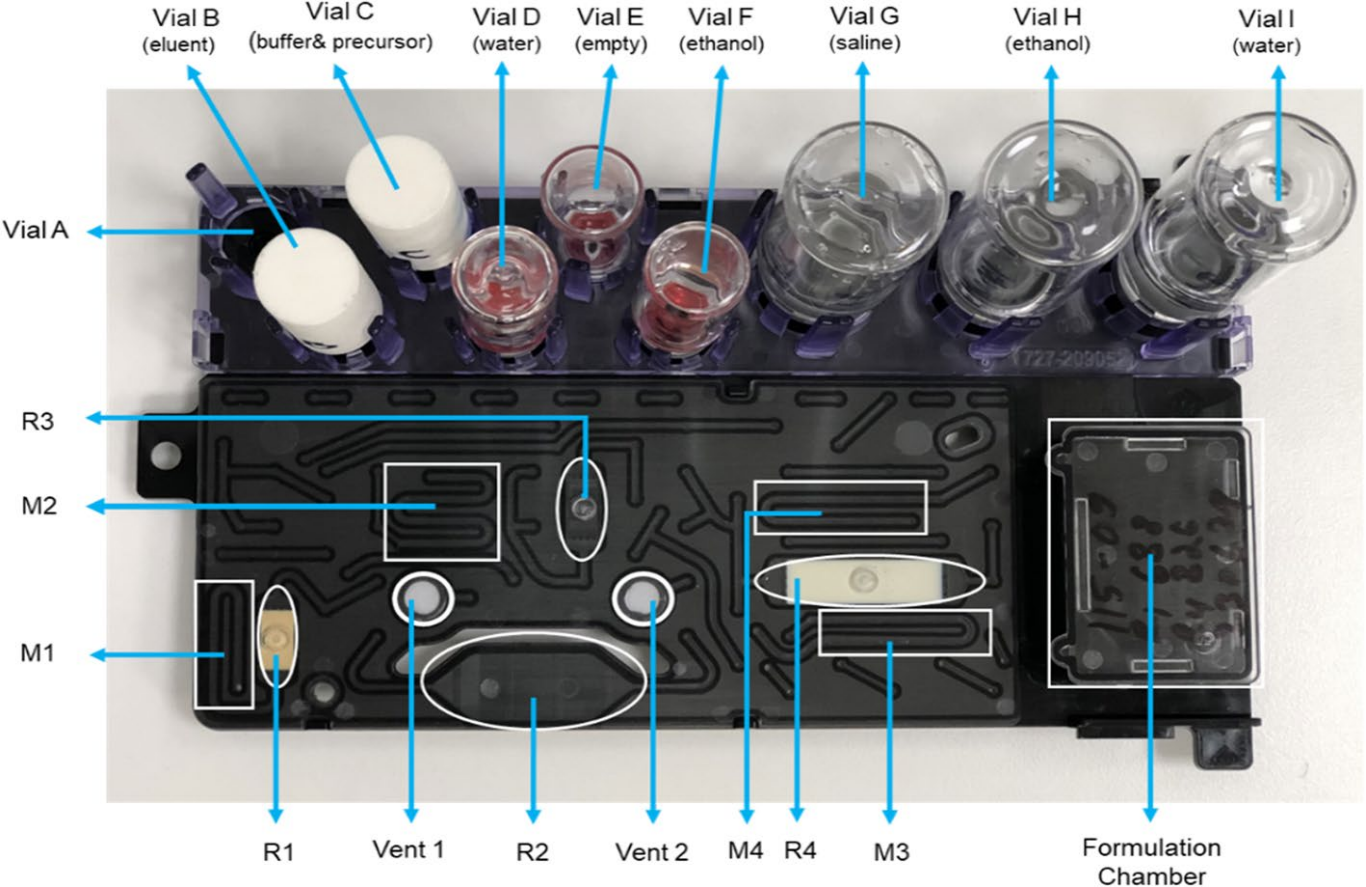
The cassette overview including all the reagents, reactors, and mixers

The microfluidic cassette-based iMiDEV™ radiosynthesizer was used to perform [^68^Ga]Ga-DOTA-TOC and [^68^Ga]Ga-FAPI-46 syntheses. The radiosynthesizer is divided into two parts: i) the synthesis box and ii) the control module. The synthesis box allows us to perform radiochemistry using the microfluidic cassette and reagents. The control module assists in performing all synthesis processes in auto mode without manual interruption. The detailed working principle of the module and cassettes was reported previously (Ovdiichuk et al. 2021; Mallapura et al. 2022). The module working principle details are provided in the Additional file 1 (SI; Working principle of iMiDEV™ radiosynthesizer; pages 7–8). An overview of the cassette, including all the reagents used for synthesis, reactors, and mixers, is shown in Fig. 5.

### Microfluidic production of [^68^Ga]Ga-FAPI-46 and [^68^Ga]Ga-DOTA-TOC

#### Optimization of elution of [^68^Ga]GaCl_3_

A pressure drop test was performed for all the cassettes to check for any leaks (Mallapura et al. 2022). The pressure drop was measured on R1 under specific pressure conditions (1 bar), using an empty vial on EV A or B, with the flow directed through R1 to the waste. These tests served to provide insights into the arrangement of the beads within the reactor chamber. When the beads in R1 were not densely packed, it resulted in reduced flow resistance. Conversely, when the beads were compactly arranged, it led to higher flow resistance. Cassettes were then selected based on the measured pressure drop on R1. Cassettes with a pressure drop of 0.2 to 0.3 bar were selected to perform trapping and elution on R1. The concentration of [^68^Ga]GaCl_3_ was performed using Chromafix PS-H + resin following the methodology proposed in previous studies (Ovdiichuk et al. 2022, 2023), and the elution technique was adapted according to the work of Mueller et al. (2012). In this approach, R1 (50 µL) was filled with Chromafix PS-H + resin to concentrate the [^68^Ga]GaCl_3_. Vial B position was utilized for eluent delivery throughout the optimization tests. We incorporated inserters and full glass vials (300 µL) during optimization to reduce the dead volume. The ^68^Ga-eluate was obtained from a ^68^Ge/^68^Ga generator in 5 mL of 0.1 M HCl. All the required reagents were carefully placed on the cassette before being clamped on the docking plate.

To explore different elution strategies, such as concentrations and volume of eluents. Two different concentrations, 0.12 M HCl in 5 M NaCl (eluent 1) and 0.15 M HCl in 5 M NaCl (eluent 2), were evaluated. Direct and reverse trapping were evaluated to improve recovery of [^68^Ga]GaCl_3_. The detailed procedure of direct and reverse trapping and elution is added in the Additional file 1 (SI; Optimization of elution of [^68^Ga]GaCl_3_; pages 8–9).

The percentage of trapping efficiency was calculated by dividing the trapped activity on R1 by the starting activity, and the percentage of recovered [^68^Ga]Ga^3+^ was calculated by dividing the collected activity by the trapped activity on the Chromafix PS-H + cartridge.

#### Optimization of radiolabeling

For radiolabeling optimization, automated synthesis was performed, and the reaction mixture was analyzed to determine the radiochemical purity. The radiolabeling reaction was also performed manually using the same volume of eluate, acetate buffer, and precursor chosen for automated radiolabeling. The reaction temperature for manual radiolabeling was set at 95 °C, and the reaction time was 5–10 min. In the first step of the automated synthesis, [^68^Ga]GaCl_3_ was trapped on R1 and filled with Chromafix PS-H + resin. After trapping [^68^Ga]GaCl_3_ on the micro cartridge (R1), it was washed with water (TraceSELECT™, for trace analysis), followed by elution of [^68^Ga]GaCl_3_ with acidified NaCl into microreactor R2 (286 µL) and simultaneous mixing with buffer and peptide mixture.

**Fig. 6:**
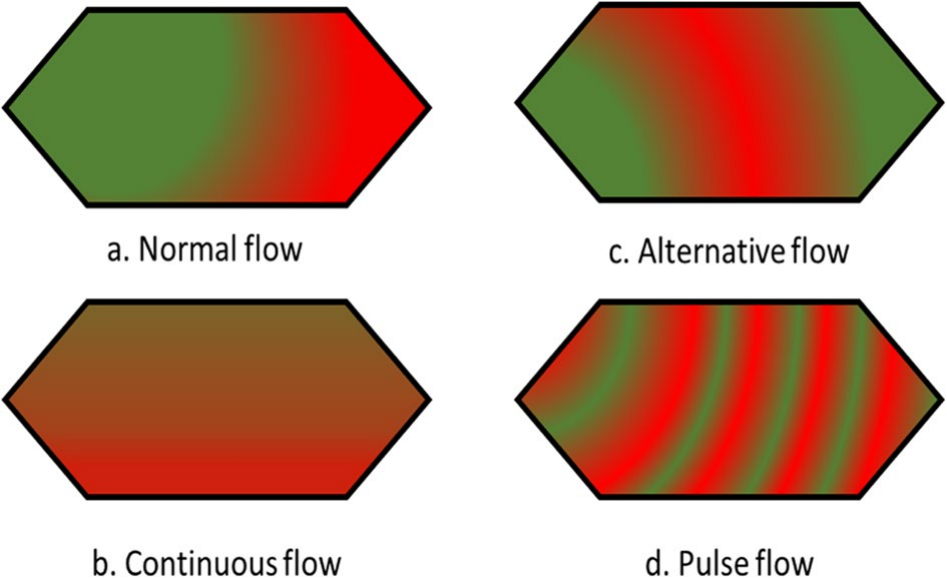
Graphical representation of distinct types of flow for reagent mixing

Different mixing techniques were applied to perform the radiolabeling. A graphical visualization of reagent mixing with different flow techniques inside reactor R2 is shown in Fig. 6. The mixing proportion of the reagent was managed with different pressures and dispensing time from eluent and buffer-peptide mixture vials.

**Fig. 7:**
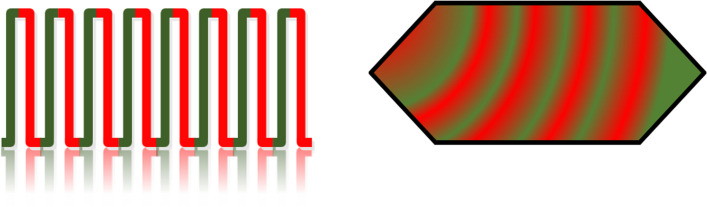
Visual representation of pulse flow and reactor 2 filling with eluent and buffer

The following mixing techniques were explored; for all these, the exact positions of the eluent (position B) and buffer-peptide mixture (position C) vials were used. The dead volume of vials B and C was 30–50 µL.

(A) Normal flow: This technique first eluted [^68^Ga]GaCl_3_ to R2 from R1 and then buffer-peptide mixture. After pushing the buffer-peptide mixture into R2, it was mixed with eluate (Fig. 6a). Then, the reaction was performed.
(B) Continuous flow: Radioactivity on R1 eluted up to mixer 2 before starting the continuous flow. Both the eluent and buffer mixture were released simultaneously to R2 through mixer 2 (Fig. 6b). Mixer 2 helps to mix reagents before entering into R2.
(C) Alternative flow: In this approach, the radioactivity eluted up to mixer 2 before starting an alternative flow. Then, a proportion of the buffer mixture was added to R2, followed by a complete elution of Ga-68, and finally, the remaining buffer mixture was added to R2. The mixing of reagents was achieved through diffusion by increasing the surface area for interaction (Fig. 6c).
(D) Pulse flow: The mixing approach was changed to continuous-alternative or pulse flow (Fig. 6d). Following this approach, the [^68^Ga]Ga^3+^ and buffer-peptide mixture was eluted part by part to preheated reactor 2 (Fig. 7). The time for opening and closing of microfluidic valves was optimized to achieve reproducibility and high radiolabeling yield. After optimization, we adapted this mixing technique for the rest of our syntheses.

After mixing, the reaction mixture was heated at 130 °C (set temperature) for 10 min, then cooled down to 40 °C. The reaction mixture was collected with sterile water to analyze radiochemical efficiency using an analytical HPLC and radio TLC scanner. After the optimization of radiolabeling, solid phase extraction (SPE) purification and formulation steps of the final product were added to the automated process. The radiolabeled product was trapped on R4 (HLB resin) while washing the reaction mixture with sterile water to separate it from the unbound ^68^Ga. After these steps, R4 was rinsed with sterile water before eluting with ethanol (56%). Finally, the product was formulated with saline and collected in a sterile vial through a sterile filter.

#### Preparation of reagents and cassette for validation run

Cassettes with a pressure drop of 0.28 to 0.30 bar were selected. The list of reagents used for the syntheses is summarized in Additional file 1: Table S6. FAPI-46 (2.26 mM) and DOTA-TOC (1.4 mM) stock solutions were prepared using water (TraceSELECT™, for trace analysis). 10 µL of the stock solution was used along with 150 µL of acetate buffer and 10 µL of sodium ascorbate (30 mg/mL) in vial C. An acidified 5 M NaCl (0.15 M HCl in 5 M NaCl) was filled in vial B for elution of ^68^Ga. Vial D was filled with 3 mL of sterile water containing sodium ascorbate (15 mg) to wash the reaction mixture from R2. Vial F was filled with 1.2 mL of ethanol (56%) for product elution from R4. Vial G was filled with 8 mL of saline (5 mg/mL sodium ascorbate) for formulation. Vial H was filled with 3 mL of ethanol for R4 preconditioning. Vial I was filled with 8 mL of sterile water containing sodium ascorbate (40 mg/mL) and used to wash R4 during SPE purification. All reagents except the precursor were the same for [^68^Ga]Ga-FAPI-46 and [^68^Ga]Ga-DOTA-TOC.

#### Radiosynthesis of ^68^Ga-labeled FAPI-46 and DOTA-TOC

**Fig. 8:**
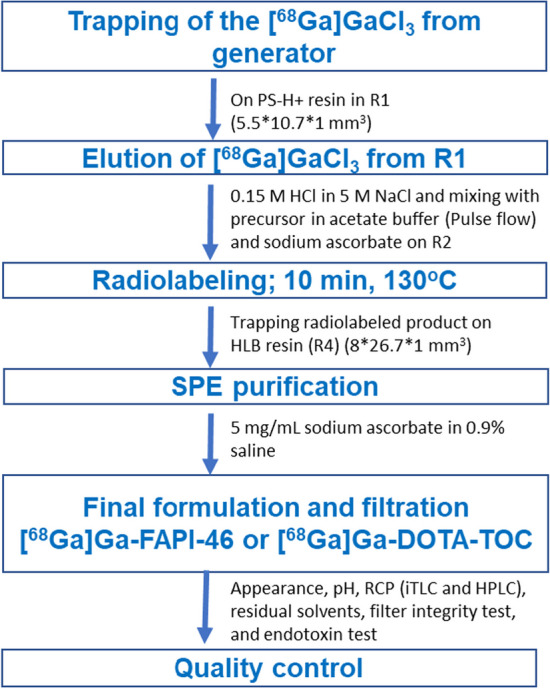
Flow chart of radiosynthesis steps

After the optimization, all the synthesis steps were performed in automatic mode except ^68^Ga -elution. All reagents were loaded into the cassette and placed in the synthesis box. The overall synthesis was performed in three steps: (a) concentration and elution of [^68^Ga]GaCl_3_, (b) radiolabeling, and (c) purification and formulation. The flow chart of the synthesis steps is shown in Fig. 8. All the synthesis steps are shown separately using iMiDEV™ supervision, and details of the radiosynthesis procedure can be found in SI (Additional file 1: Figure S7–S14; pages 10–14).

**Fig. 9:**
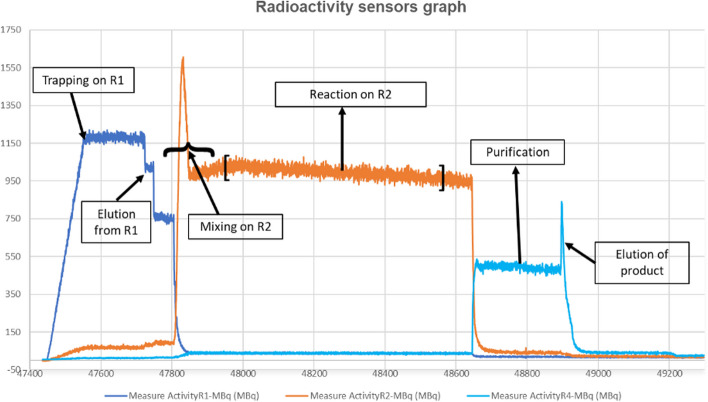
Radioactivity sensors graph of the complete radiosynthesis

After the end of the synthesis, quality control tests were performed. The radioactivity sensors graph from the complete synthesis is shown in Fig. 9. The radiochemical yield was calculated by dividing the decay-corrected product activity by the initial activity employed in the synthesis, which was subtracted from the residual initial activity after the synthesis.

The apparent molar activity of [^68^Ga]Ga-FAPI-46 and [^68^Ga]Ga-DOTA-TOC was analyzed using the same method as the analytical HPLC method used for radiochemical purity determination, and was calculated using the calibration curve for both [^68^Ga]Ga-FAPI-46 and [^68^Ga]Ga-DOTA-TOC.

### Quality control

The final product's appearance was checked by visually inspecting the final product vial after the end of synthesis. The pH of the final product was determined using pH paper (pH 2–9, MQuant®, Merck). Radiochemical purity and stability were determined using analytical HPLC (Agilent) and iTLC from a LabLogic Scan-RAM TLC system. Analytical HPLC has a quaternary pump G1311A, auto-injector, ultraviolet (UV) detector G1314D, and radio detector. For [^68^Ga]Ga-FAPI-46, a reversed-phase column C18, 5 µm, 4.6*150 mm (Waters Atlantis) was used as the stationary phase and gradient mobile phase of 0.1% trifluoroacetic acid (TFA) in acetonitrile (solvent A) and 0.1% TFA in H_2_O (solvent B); 0–8 min, 13–87% solvent A; 8–10 min, 87–13% solvent A; 10–14 min, 13% solvent A. The flow rate was 1.2 mL/min, and the UV wavelength was 220 nm. For [^68^Ga]Ga-DOTA-TOC, a reversed-phase column XBridge C18, 5 µm, 4.6*150 mm was used as the stationary phase and gradient mobile phase of 0.1% TFA in acetonitrile (solvent A) and 0.1% TFA in H_2_O (solvent B); 0–10 min, 13%–100% solvent A; 10–12 min, 100–87% solvent A; 12–16 min, 13% solvent A. The flow rate was 1.2 mL/min, and the UV wavelength was 260 nm. For iTLC, glass microfiber chromatography paper impregnated with silica gel (SG) was used as a stationary phase, and 50% of 1 M ammonium acetate and 50% methanol (1:1) were used as mobile phase for both radiotracers. Radionuclide identity was determined by measuring the half-life of the radionuclide. Residual solvent analysis was performed using an Agilent (model 6850) gas chromatography. Endotoxin tests were performed using the Endosafe® Nextgen-PTS Kinetic Reader (Charles River Laboratories, USA). Filter integrity test performed using the custom-made bubble point tester by DM automation, Sweden.

### Supplementary Information

**Additional file1. Figure S1**: TLC chromatogram of [^68^Ga]Ga-FAPI-46 synthesis at EOS; **Figure S2**: TLC chromatogram of [^68^Ga]Ga-DOTA-TOC synthesis at EOS; **Figure S3**: HPLC chromatogram of [^68^Ga]Ga-FAPI-46 synthesis; **Figure S4**: HPLC chromatogram of [^68^Ga]Ga-DOTA-TOC synthesis; **Figure S5**: Reaction schematic of [^68^Ga]Ga-DOTA-TOC synthesis; **Figure S6:** Reaction schematic of [^68^Ga]Ga-FAPI-46 synthesis; **Figure S7**: Reverse trapping of [^68^Ga]GaCl_3_ on R1; **Figure S8**: Elution of [^68^Ga]GaCl_3_ from R1 to R2 using vial B; **Figure S9**: Addition of buffer into R2 from vial C; **Figure S10**: Washing of R2 from vial D and trapping of labeled product on R4; **Figure S11**: Washing of R4 with water from vial I towards HPLC waste; **Figure S12**: Elution of product with 56% ethanol from R4 using vial F; **Figure S13**: Formulation of product in the formulation chamber with 0.9% saline using vial G; **Figure S14**: Collection of product from formulation chamber through sterile filtration; **Table S1**: Details of [^68^Ga]Ga-FAPI-46 synthesis method validation performed at Nancyclotep (France); **Table S2**: Radiochemical yield details of [^68^Ga]Ga-FAPI-46 using a pulse flow approach (n=13) with iMiDEV^TM^ module; **Table S3**: Radioactivity distribution of the complete radiosyntheses of [^68^Ga]Ga-FAPI-46 and [^68^Ga]Ga-DOTA-TOC; **Table S4**: Residual activity distribution on the cassette after the complete syntheses of [^68^Ga]Ga-FAPI-46 and [^68^Ga]Ga-DOTA-TOC; **Table S5**. Comparison of [^68^Ga]Ga-FAPI-46 synthesis using iMiDEV™ module versus other conventional synthesizers; **Table S6:** List of reagents used for [^68^Ga]Ga-FAPI-46 and [^68^Ga]Ga-DOTA-TOC syntheses.

## Data Availability

The datasets used and/or analyzed during the current study are available from the corresponding author on reasonable request.

## Abbreviations

AMA: Apparent molar activity
d.c.: Decay corrected.
DOD: Dose on demand
DOTA: 2,2′,2′′,2′′′-(1,4,7,10-Tetraazacyclododecane-1,4,7,10-tetrayl)tetraacetic acid
GC: Gas chromatography
HPLC: High performance liquid chromatography
PET: Positron emission tomography
Ph. Eur.: European pharmacopoeia
Q.C.: Quality control
RCP: Radiochemical purity
RCY: Radiochemical yield
SPE: Solid phase extraction
TFA: Trifluoroacetic acid
TLC: Thin layer chromatography
UV: Ultraviolet
