# Supplementary material for: Microfluidic-based production of [68Ga]Ga-FAPI-46 and [68Ga]Ga-DOTA-TOC using the cassette-based iMiDEV™ microfluidic radiosynthesizer
Source: EJNMMI Radiopharm Chem. 2023 Dec 13;8:42. doi: 10.1186/s41181-023-00229-9 (PMC10719436; doi:10.1186/s41181-023-00229-9)
Supplement: Supplementary file 1 — Additional file1. Figure S1: TLC chromatogram of [68Ga]Ga-FAPI-46 synthesis at EOS; Figure S2: TLC chromatogram of [68Ga]Ga-DOTA-TOC synthesis at EOS; Figure S3: HPLC chromatogram of [68Ga]Ga-FAPI-46 synthesis; Figure S4: HPLC chromatogram of [68Ga]Ga-DOTA-TOC synthesis; Figure S5: Reaction schematic of [68Ga]Ga-DOTA-TOC synthesis; Figure S6: Reaction schematic of [68Ga]Ga-FAPI-46 synthesis; Figure S7: Reverse trapping of [68Ga]GaCl3 on R1; Figure S8: Elution of [68Ga]GaCl3 from R1 to R2 using vial B; Figure S9: Addition of buffer into R2 from vial C; Figure S10: Washing of R2 from vial D and trapping of labeled product on R4; Figure S11: Washing of R4 with water from vial I towards HPLC waste; Figure S12: Elution of product with 56% ethanol from R4 using vial F; Figure S13: Formulation of product in the formulation chamber with 0.9% saline using vial G; Figure S14: Collection of product from formulation chamber through sterile filtration; Table S1: Details of [68Ga]Ga-FAPI-46 synthesis method validation performed at Nancyclotep (France); Table S2: Radiochemical yield details of [68Ga]Ga-FAPI-46 using a pulse flow approach (n=13) with iMiDEVTM module; Table S3: Radioactivity distribution of the complete radiosyntheses of [68Ga]Ga-FAPI-46 and [68Ga]Ga-DOTA-TOC; Table S4: Residual activity distribution on the cassette after the complete syntheses of [68Ga]Ga-FAPI-46 and [68Ga]Ga-DOTA-TOC; Table S5. Comparison of [68Ga]Ga-FAPI-46 synthesis using iMiDEV™ module versus other conventional synthesizers; Table S6: List of reagents used for [68Ga]Ga-FAPI-46 and [68Ga]Ga-DOTA-TOC syntheses. [file 41181_2023_229_MOESM1_ESM.docx]

**Supplementary information**

**Microfluidic-based production of [^68^Ga]Ga-FAPI-46 and [^68^Ga]Ga-DOTA-TOC using the cassette-based iMiDEV™ microfluidic radiosynthesizer.**

**Hemantha Mallapura^1,*^, Olga Ovdiichuk^2^, Emma Jussing^3^, Tran A Thuy^3^, Camille Piatkowski^4^, Laurent Tanguy^4^, Charlotte Collet-Defossez^2,5^, Bengt Långström^6^, Christer Halldin^1^, and Sangram Nag^1^**

^1^ Department of Clinical Neuroscience, Center for Psychiatry Research, Karolinska Institutet, and Stockholm County Council, SE-17176, Stockholm Sweden; [hemantha.mallapura@ki.se](mailto:hemantha.mallapura@ki.se) (H.M.); [christer.halldin@ki.se](mailto:christer.halldin@ki.se) (C.H.); [sangram.nag@ki.se](mailto:sangram.nag@ki.se) (S.N.)

^2^ Nancyclotep, Molecular Imaging Platform, 5 rue du Morvan, F-54500 Vandoeuvre les Nancy, France; [o.ovdiichuk@nancyclotep.com](mailto:o.ovdiichuk@nancyclotep.com) (O.O.)

^3^ Department of Oncology and Pathology, Karolinska Institutet, SE-17177 Stockholm, Sweden; and De-partment of Radiopharmacy, Karolinska University Hospital, SE-17176, Stockholm Sweden; [emma.jussing@ki.se](mailto:emma.jussing@ki.se) (E.J.); [thuy.tran@regionstockholm.se](mailto:thuy.tran@regionstockholm.se) (T.T.)

^4^ PMB-Alcen, Route des Michels CD56, F-13790 Peynier, France; [ltanguy@pmb-alcen.com](mailto:ltanguy@pmb-alcen.com) (L.T.); [cpiatkowski@pmb-alcen.com](mailto:cpiatkowski@pmb-alcen.com) (C.P.)

^5^ Université de Lorraine, Insert, IADI, F-54000 Nancy, France; [charlotte.collet@univ-lorraine.fr](mailto:charlotte.collet@univ-lorraine.fr) (C.C.)

^6^ Department of Chemistry, Uppsala University, Uppsala 75123, Sweden**;** [bengt.langstrom@kemi.uu.se](mailto:bengt.langstrom@kemi.uu.se) (B.L.)

Correspondence: hemantha.mallapura@ki.se

**Corresponding author:**

Hemantha Mallapura

Department of Clinical Neuroscience

Center for Psychiatry Research

Karolinska Institutet, and Stockholm County Council

SE-171 76 Stockholm, Sweden

E-mail: hemantha.mallapura@ki.se

Phone: +46-769084212

**Figure S1.** TLC chromatogram of [^68^Ga]Ga-FAPI-46 synthesis at EOS


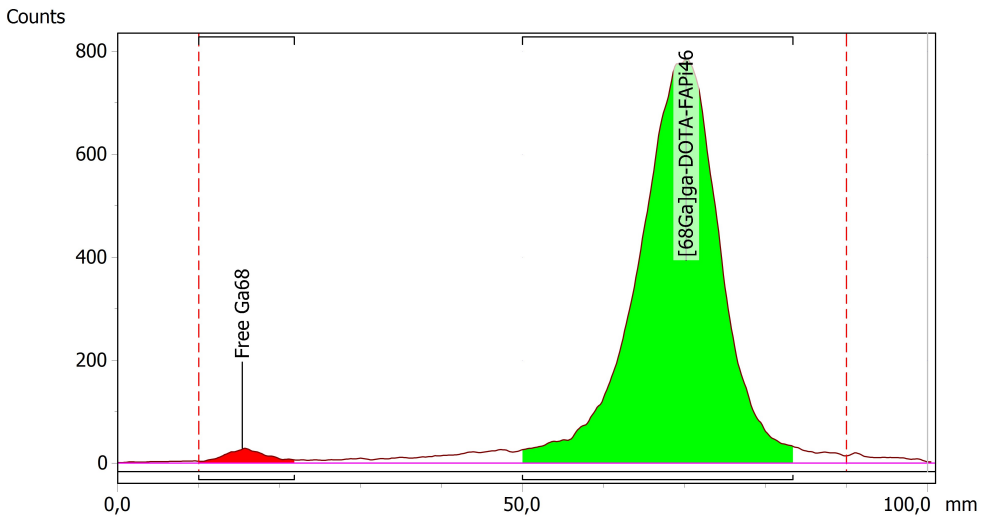

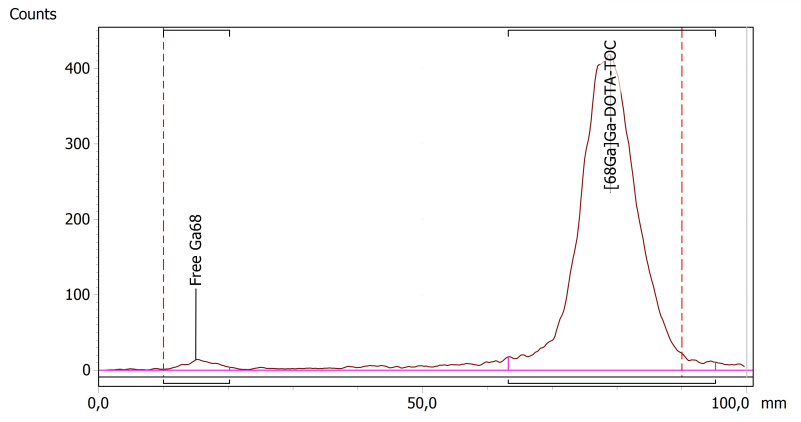


**Figure S2.** TLC chromatogram of [^68^Ga]Ga-DOTA-TOC synthesis at EOS


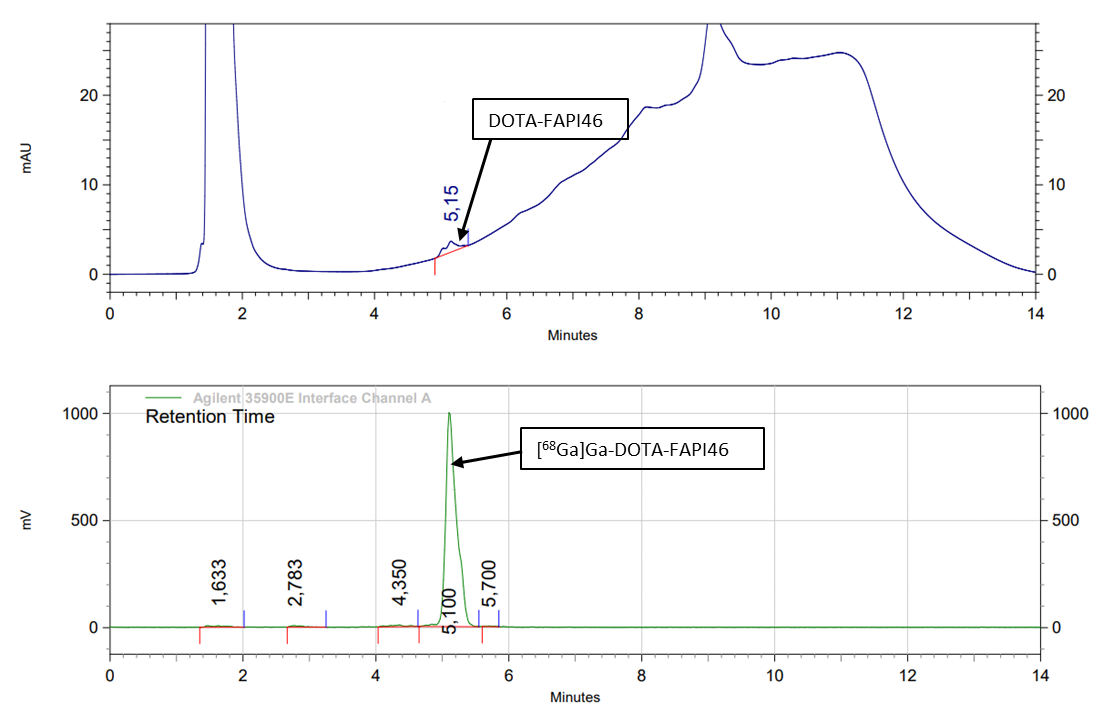


**Figure S3.** HPLC chromatogram of [^68^Ga]Ga-FAPI-46 synthesis

**Figure S4.** HPLC chromatogram of [^68^Ga]Ga-DOTA-TOC synthesis


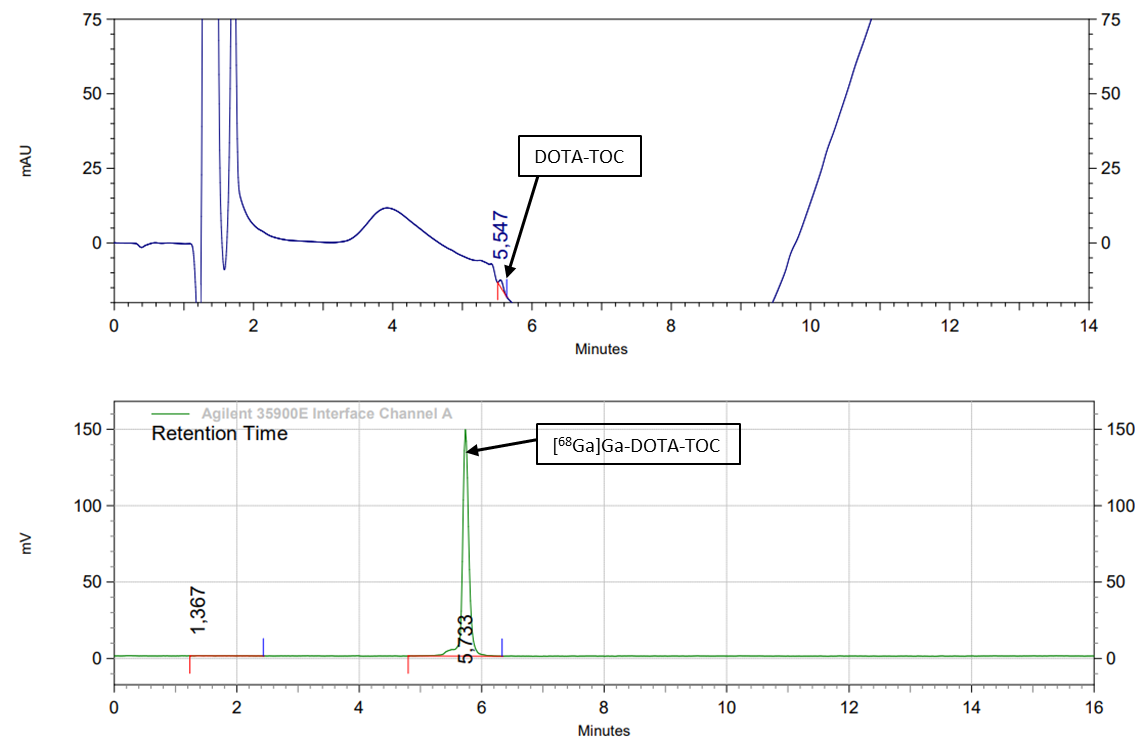

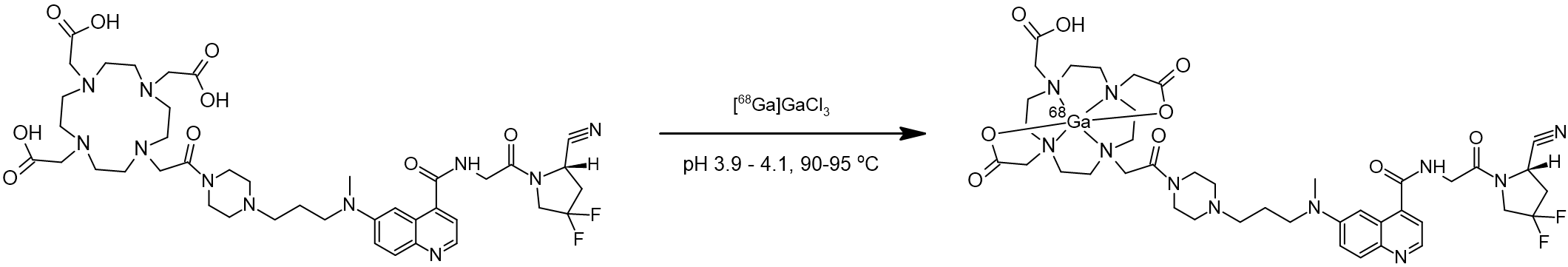


**Figure S6.** Reaction schematic of [^68^Ga]Ga-FAPI-46 synthesis

**Figure S5.** Reaction schematic of [^68^Ga]Ga-DOTA-TOC synthesis


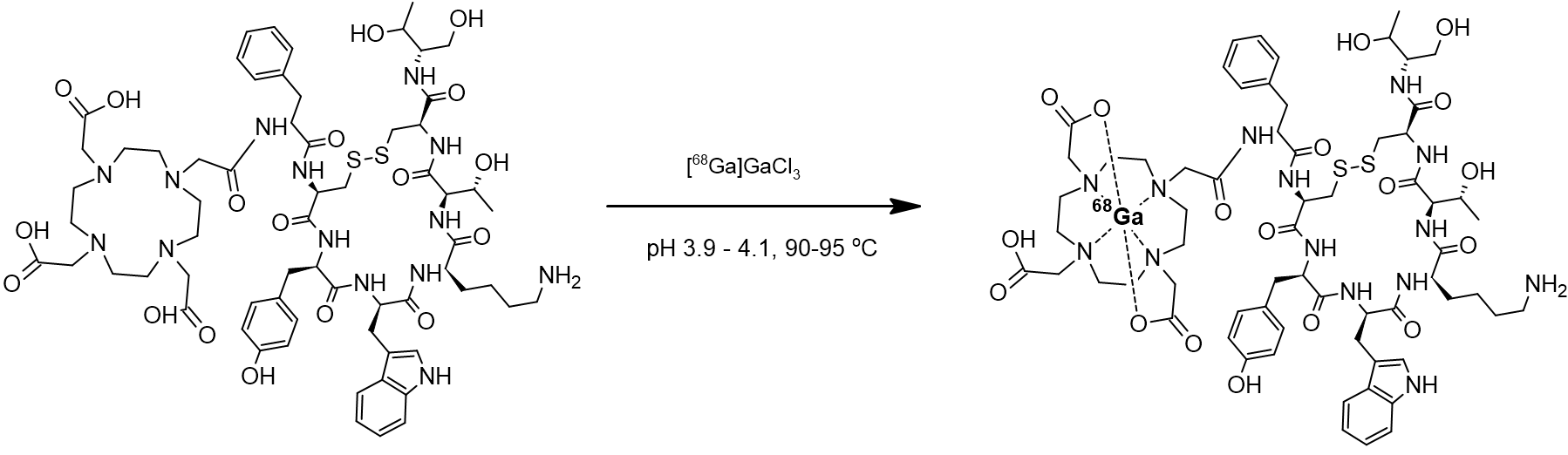


**Table S1.** Details of [^68^Ga]Ga-FAPI-46 synthesis method validation performed at Nancyclotep (France)

| Syn No | Radiochemical yield_dc_ (%) | Residual activity on the cassette  (%) | Radiochemical purity (%) | pH |
| --- | --- | --- | --- | --- |
| FAPI46-0601 | 48.5 | 12.4 | 98.2 | 5.5 |
| FAPI46-0901 | 33.7 | 12.1 | 97.6 | 5.5 |
| FAPI46-1901 | 43.4 | 7.8 | 97.6 | 5.5 |
| Average | **41.8** | **10.8** | **97.8** |  |
| Std dev | **6.1** | **2.1** | **0.3** |  |

**Table S2.** Radiochemical yield details of [^68^Ga]Ga-FAPI-46 using a pulse flow approach (n=13) with iMiDEV™ module

| Syn No | Yield(dc) % |
| --- | --- |
| 130 | 46 |
| 131 | 53 |
| 132 | 52 |
| 135 | 46 |
| 146 | 63 |
| 147 | 52 |
| 148 | 60 |
| 150 | 50 |
| 151 | 49 |
| 153 | 47 |
| 155 | 47 |
| 160 | 47 |
| 161 | 51 |
| Average | **51** |
| Std dev | **5.1** |

**Table S3.** Radioactivity distribution of the complete radiosyntheses of [^68^Ga]Ga-FAPI-46 and [^68^Ga]Ga-DOTA-TOC

* - [^68^Ga]Ga-DOTA-TOC

| Syn No | Product (%) | Syn waste  (%) | Filter  (%) | Cassette  (%) |
| --- | --- | --- | --- | --- |
| 161 | 51 | 37 | 1.8 | 7.8 |
| 167* | 54.7 | 32.7 | 4.3 | 8 |
| 168* | 46 | 43 | 2 | 7.8 |
| Average | 50.6 | 37.6 | 2,7 | 7.9 |
| Std dev | 3.6 | 4.2 | 1.1 | 0.1 |

**Table S4.** Residual activity distribution on the cassette after the complete syntheses of [^68^Ga]Ga-FAPI-46 and [^68^Ga]Ga-DOTA-TOC

| Syn no | R1 (%) | R2 (%) | R3 (%) | R4 (%) | Vents (%) | Formulation Chamber (%) | Total on the cassette (%) |
| --- | --- | --- | --- | --- | --- | --- | --- |
| 161 | 1.6 | 0.22 | 0.33 | 4.88 | 0.68 | 0.4 | 8.11 |
| 167* | 1.1 | 1.47 | 1.24 | 2.35 | 1.1 | 0.53 | 7.79 |
| 168* | 1.85 | 1.42 | 0.06 | 2.5 | 1.5 | 0.45 | 7.78 |
| Average | **1.5** | **1.0** | **0.5** | **3.2** | **1.1** | **0.5** | 7.9 |
| Std Dev | **0.3** | **0.6** | **0.5** | **1.2** | **0.3** | **0.1** | 0.2 |

* - [^68^Ga]Ga-DOTA-TOC

| **Test** | **Acceptance criteria** | **iMiDEV™** | **MLPT*** | **EasyOne**** |
| --- | --- | --- | --- | --- |
| Appearance | Clear or slightly yellow and free of particles | Conform | Conform | Conform |
| pH | 4.0–8.0 | 5.83 ± 0.1 | 5.3 ± 0 | 6.5 |
| Total radiochemical purity^1^ (%) | ≥91 | 96.0±0.40 | 98.3±1.20 | >99.4% |
| Filter integrity (bar) | ≥3.5 | 4.1 ± 0.1 | 4.2 ± 0. | >3.2 |
| Bacterial endotoxins (EU/mL) | <17.5 | <5.0 | <5.0 | <5.0 |
| Ethanol (%) G.C. | <10 | 6.7± 0.2 | 6.4 ± 0.45 | - |
| Radiochemical stability after 3 hrs (%) | ≥91% | 95.8±0.80 | 95±0.02 | - |
| Sterility | Sterile | - | Pass | Pass |
| Synthesis time (min) | Not specified | < 30.0 | 17.0 | 12.0 |
| Apparent molar activity (GBq/µmol) | Not specified | 30±11 | 10±1.7 | - |
| Radiochemical Yield_dc_ (%) | Not specified | 44±5 | 69±4 | 92±5 |
| Number of synthesis (n) | Not specified | 3 | 4 | 3 |
| Precursor (µg) | Not specified | 20 | 50 | 50 |

**Table S5.** Comparison of [^68^Ga]Ga-FAPI-46 synthesis using iMiDEV™ module versus other conventional synthesizers

***-** Modular-Lab Pharmtracer from Eckert and Ziegler; **- Trasis

**Methods**

***4.1. Microfluidic cassette-based iMiDEV™ radiosynthesizer***

**Working principle of iMiDEV™ radiosynthesizer**

The docking plate has thirty-four microfluidic valves (MVFs) and nine electrovalves (EVs) in the synthesis box. MVFs are operated by charging compressed air, and electrovalves are pressurized with helium/nitrogen gas and actuated to transfer reagents. The clamping system presses the cassette against all these valves. Radioactivity sensors are integrated into the clamping plate with the respective positions of the reactors (R1, R2, R3 and R4). When the microfluidic cassette is clamped, the sensors are precisely placed on top of the reactors to monitor the radioactivity inside the cassette during the radiosynthesis process. The radioactive sensors measure the approximate radioactivity amount corresponding to coefficient values. The Peltier thermoelectric module is used to heat and cool reactor 2, and the temperature range is from 20 to 130 °C.

A detailed description of the microfluidic cassettes as described in our previous articles. An overview of the cassette is shown using iMiDEV™ supervision in Figure 1. The microfluidic channels are connected using microfluidic valves with respective reactors and electrovalves (A to G) to transfer reagents (100 µL to 10 mL) using helium/nitrogen gas (0.1 to 1.9 bar). There are three reactors (R1; 50 µL, R2; 286 µL and R3; 50 µL) for room- and high-temperature reactions, particularly R1 and R3, which are used for room-temperature reaction on solid supports, radionuclide concentration and sometimes for purification, and another reactor (R4; 200 µL) dedicated to SPE purification. However, it can be used for room-temperature reactions on solid support as well. The 12 mL formulation chamber facilitates the formulation of the final product before sterile filtration. We utilized R1, R2 and R4 for this study. Reactor R1 was filled with the strong cation exchange resin PS-H^+^ for [^68^Ga]Ga^3+^ trapping and elution, whereas reactor R4 was filled with C18/hydrophilic-lipophilic balanced (HLB) resin for solid-phase extraction (SPE) purification. The design and structure of the cassette are the same as those used in previous studies, and resins are changed in reactors (R1, R2 and R4) as per our applications.

All microfluidic cassettes were examined before being used in radiosynthesis. The filling of resin on the reactors was examined visually. A pressure drop test was performed for all cassettes before being used for synthesis to check for eventual leakages and estimate flow restrictions on R1 and R4. The pressure drop test is an important parameter related to the density of beads in R1 and R4. The density of beads, especially in R1, considerably influences the trapping and elution performances. High beads density usually means poorer trapping-elution performance.

Automated radiolabeling was performed using the iMiDEV™ microfluidic radiosynthesizer. The microfluidic cassette has vials for reagents, micro cartridges, and reactors for trapping radionuclides, radiolabeling, and purification. All synthesis steps were conducted in auto mode, except for the elution step, without manual intervention. The elution of radioactivity on R1 required manual handling.

***4.2. Microfluidic production of [^68^Ga]Ga-FAPI-46 and [^68^Ga]Ga-DOTA-TOC***

**Optimization of elution of [^68^Ga]GaCl_3_**

For direct trapping and elution, [68Ga]GaCl3 was trapped on R1 by opening MFVs 3 and 6 towards the waste using 0.75 bar and 1 bar of vector gas (helium gas), respectively. After trapping, R1 was rinsed with 2 mL of TraceSELECT™ water and then flushed with helium using 1.5 bar pressure for 30 seconds. Subsequently, the trapped [^68^Ga]GaCl_3_ was eluted to R2 with the vial in B position using 200 mbar pressure by opening MFVs 14, 11, 13, 12, 7, and 1.

A range of 140-200 µL of eluent was used for elution due to the volume of the R2 chamber (286 µL). Two vents are positioned before and after R2, close to MVFs 11 and 14, to facilitate the removal of air bubbles during the filling of R2 (Figure 1). Following elution, R2 was washed with 3 mL of TraceSELECT™ water through the HPLC waste line by opening MFVs 20, 18, 13, 12, and 9 from vial D.

For the reverse trapping and elution process, we explored different paths for trapping while maintaining a consistent path for elution. [^68^Ga]GaCl_3_ trapping was achieved on R1 by opening MFVs 8, 7, 5, 16, and 21. Subsequently, R1 was thoroughly washed using TraceSELECT™ water, following the same path as the trapping process. The vial E position was blocked on the cassette to facilitate reverse trapping. For elution, [^68^Ga]GaCl_3_ was released from R1 through the same path used for direct elution.

**Quality control at Nancyclotep radiochemistry lab:** The chemical and radiochemical purities and identity of the [^68^Ga]Ga-FAPI-46 were determined by analyzing an aliquot of [^68^Ga]Ga-FAPI-46 on HPLC carried out on a Shimadzu LC-40 system equipped with an SPD-M40 photodiode array (PDA) detector and a radio HPLC detector (Herm LB500 with Flumo detector, Berthold, Bad Wildbad, Germany) controlled by Labsolutions Software (Kyoto, Japan). Chromatographic separations were performed by gradient elution: 0-12.5 min; 13% solvent A, 12.5-17 min; 100% solvent A, 17-20 min; 13% solvent A at 1.5 mL/min flow rate. The UV absorbance was measured at a wavelength of 220 nm. The pH of the final product was measured using pH paper (pH 2.0-9.0, MQuant®, Merck).

**Table S6.** List of reagents used for [^68^Ga]Ga-FAPI46 and [^68^Ga]Ga-DOTA-TOC syntheses.

| **Vial position** | **Reagents filled** |
| --- | --- |
| B | 200 µL of 0.15 M HCl in 5 M NaCl |
| C | 10 µL (20 µg) of precursor +150 µL of acetate buffer + 10 µL (300 µg) of sodium ascorbate |
| D | 3 mL of sterile water (15 mg sodium ascorbate) |
| F | 1.2 mL of 56% of ethanol in sterile water |
| G | 8 mL of saline (5 mg/mL sodium ascorbate) |
| H | 3 ml of ethanol |
| I | 8 mL of sterile water (5 mg/mL sodium ascorbate) |

**Figure S7.** Reverse trapping of [^68^Ga]GaCl_3_ on R1


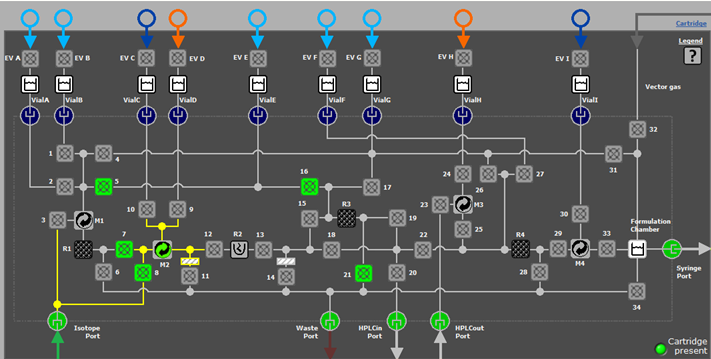


**Figure S8.** Elution of [^68^Ga]GaCl_3_ from R1 to R2 using vial B


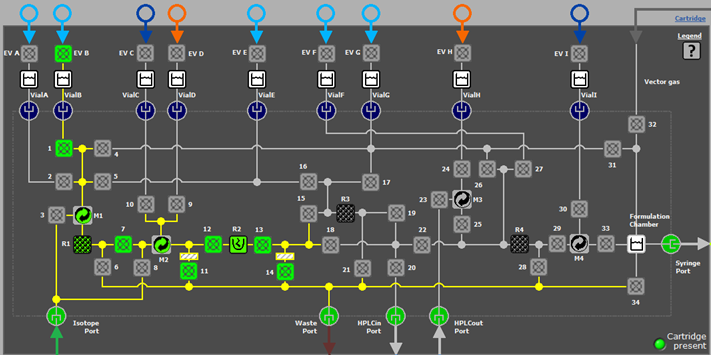


**Figure S9.** Addition of buffer into R2 from vial C


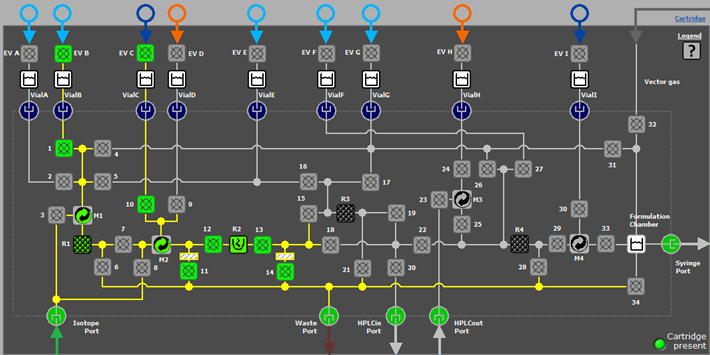


**Figure S10.** Washing of R2 from vial D and trapping of labeled product on R4


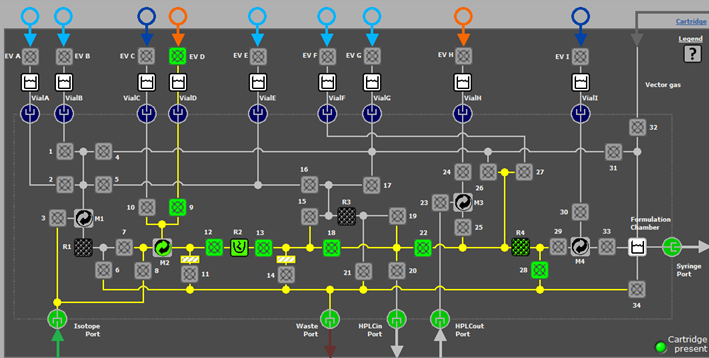


**Figure S11.** Washing of R4 with water from vial I towards HPLC waste


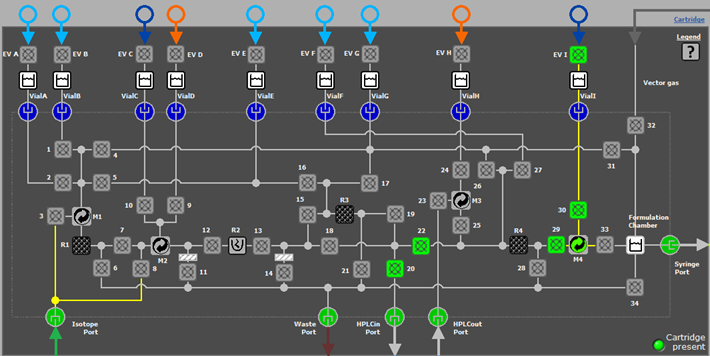


**Figure S12.** Elution of product with 56% ethanol from R4 using vial F


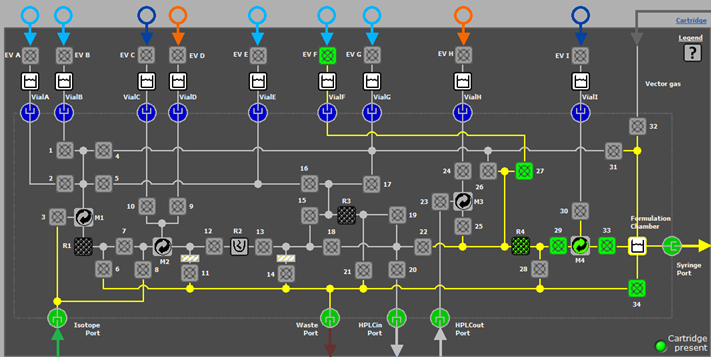


**Figure S13.** Formulation of product in the formulation chamber with 0.9% saline using vial G


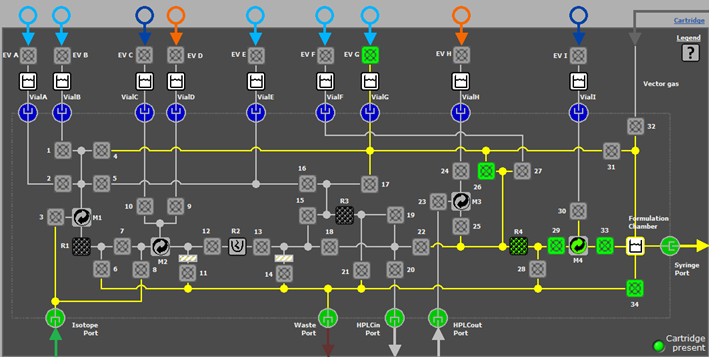

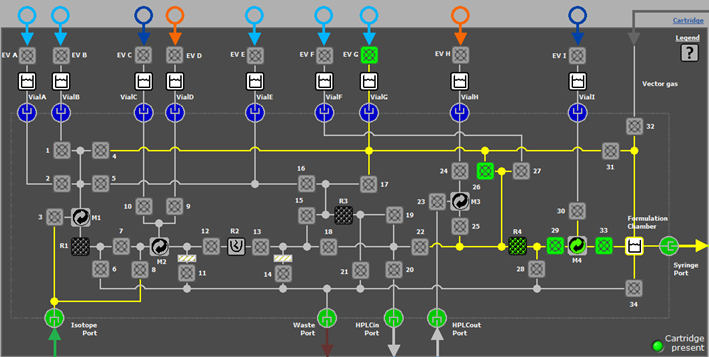


**Figure S14.** Collection of product from formulation chamber through sterile filtration

**Radiosynthesis of ^68^Ga-labeled FAPI-46 and DOTA-TOC**

**a) Concentration, elution of [^68^Ga]GaCl_3_ and reagent mixing:** Ga-68 eluate was trapped in R1 and eluted in the reverse direction. The eluate was trapped by opening MFVs 8, 7, 5, 16, and 21, respectively. After [^68^Ga]GaCl_3_ was concentrated on R1, it was rinsed with 2 mL of TraceSELECT™ water in the same direction. [^68^Ga]GaCl_3_ was recovered from R1 using 200 µL of eluent (0.15 M HCl in 5 M NaCl) by opening MFVs 1, 7, 11, 12, 13, and 14 and in the meantime, it was mixed with buffer and peptide (170 µL) following pulse flow (Figure 3) from vials B and C using 200 mbar and 130 mbar pressure. Once the radioactivity began to be released from R1 and approached mixer M2, the buffer mixture was concurrently released by opening V10. Subsequently, the eluent and buffer-peptide mixture were mixed through pulse flow from vials B and C. Radiolabeling was performed at the end of pulse-flow.

**b) Radiolabeling of FAPI-46 and DOTA-TOC:** When the eluent and buffer mixture were mixed through pulse flow in R2, the approximate pH of the reaction mixture was 4. The reaction mixture was heated at 130 °C for 10 min by closing all the MFVs. During the reaction, R4 was preconditioned with 3 mL of ethanol (vial H) by opening MVFs 24, 25, and 28, and rinsed with 4 mL of water from vial I (5 mg/mL sodium ascorbate) by opening MVFs 20, 22, 29, and 30. After the reaction, R2 was cooled down to 40 °C, and the crude mixture was sent for further purification and formulation.

**c) Purification and formulation:** The crude mixture was trapped on R4 and washed with 3 mL of water (5 mg/mL sodium ascorbate) from vial D by opening MFVs 28, 22, 18, 13, 12, and 9. The radiolabeled product was retained in R4 (HLB), and unbound ^68^Ga was passed into the waste. After this step, R4 was washed with water (5 mg/mL sodium ascorbate) from vial I by opening valves 20, 22, 29, and 30. Then, the product was eluted with 56% ethanol in water to the formulation chamber from vial F by opening MFVs 34, 33, 29, and 27. The product was diluted with 8 mL of 0.9% saline (5 mg/mL sodium ascorbate) from vial G into the formulation chamber by opening MVFs 26, 29, 33, and 34. Finally, the product was filtered through a sterile filter and collected in a sterile vial.
